# The metabolic demands of cancer cells are coupled to their size and protein synthesis rates

**DOI:** 10.1186/2049-3002-1-20

**Published:** 2013-11-07

**Authors:** Sonia C Dolfi, Leo Li-Ying Chan, Jean Qiu, Philip M Tedeschi, Joseph R Bertino, Kim M Hirshfield, Zoltán N Oltvai, Alexei Vazquez

**Affiliations:** 1 Department of Medicine, Rutgers Cancer Institute of New Jersey, Rutgers, The State University of New Jersey, New Brunswick, NJ, USA; 2 Department of Technology R&D, Nexcelom Bioscience LLC, Lawrence, MA, USA; 3 Department of Pathology, University of Pittsburgh School of Medicine, Pittsburgh, PA, USA; 4 Department of Radiation Oncology, Rutgers Cancer Institute of New Jersey, Rutgers, The State University of New Jersey, New Brunswick, NJ, USA; 5 Center for Systems Biology, Rutgers Cancer Institute of New Jersey, Rutgers, The State University of New Jersey, New Brunswick, NJ, USA

**Keywords:** Cancer metabolism, Cell size, Proliferation rate, Mesenchymal cells, Cholesterol synthesis inhibitors

## Abstract

**Background:**

Although cells require nutrients to proliferate, most nutrient exchange rates of the NCI60 panel of cancer cell lines correlate poorly with their proliferation rate. Here, we provide evidence indicating that this inconsistency is rooted in the variability of cell size.

**Results:**

We integrate previously reported data characterizing genome copy number variations, gene expression, protein expression and exchange fluxes with our own measurements of cell size and protein content in the NCI60 panel of cell lines. We show that protein content, DNA content, and protein synthesis per cell are proportional to the cell volume, and that larger cells proliferate slower than smaller cells. We estimate the metabolic fluxes of these cell lines and show that their magnitudes are proportional to their protein synthesis rate and, after correcting for cell volume, to their proliferation rate. At the level of gene expression, we observe that genes expressed at higher levels in smaller cells are enriched for genes involved in cell cycle, while genes expressed at higher levels in large cells are enriched for genes expressed in mesenchymal cells. The latter finding is further corroborated by the induction of those same genes following treatment with TGFβ, and the high vimentin but low E-cadherin protein levels in the larger cells. We also find that aromatase inhibitors, statins and mTOR inhibitors preferentially inhibit the *in vitro* growth of cancer cells with high protein synthesis rates per cell.

**Conclusions:**

The NCI60 cell lines display various metabolic activities, and the type of metabolic activity that they possess correlates with their cell volume and protein content. In addition to cell proliferation, cell volume and/or biomarkers of protein synthesis may predict response to drugs targeting cancer metabolism.

## Background

Cancer cells exhibit metabolic phenotypes that distinguish them from normal tissue cells, in particular an increased activity of metabolic pathways necessary for cell growth [1,2]. In turn, accumulating evidence indicates that major oncogenes, for example, Ras and Myc, positively regulate metabolic pathways that are upregulated in cancer cells [2-6], whereas tumor suppressors like p53 negatively regulate them [7,8]. However, a parallel understanding of cancer metabolism from basic principles is also needed, particularly in cases where the regulatory mechanisms contradict what is expected from efficiency. A good example is the Warburg effect [9]: the observation of a high glycolysis rate under normal oxygen conditions (aerobic glycolysis). While we have some understanding of the regulatory mechanisms activating glycolysis, it is not clear why the less efficient glycolysis (two ATP molecules per glucose molecule) is preferred to the more efficient oxidative phosphorylation (oxidative phosphorylation (OxPhos), 32 ATP molecules per glucose molecule).

The yield of ATP per glucose molecule has generally been used to compare the efficiency of glycolysis and OxPhos. However, cell metabolism can also be constrained by the solvent capacity of the cell cytoplasm, that is, the maximum amount of macromolecules that can occupy the intracellular space [10,11]. The simultaneous consideration of glucose uptake and solvent capacity provides a theoretical explanation for the Warburg effect [1]: at low glucose uptake rates when the glucose uptake capacity is the limiting factor, mitochondrial respiration is indeed the most efficient pathway for ATP generation. Above a threshold glucose uptake rate, however, the solvent capacity becomes the limiting factor, resulting in gradual activation of aerobic glycolysis and slight decrease of mitochondrial respiration. Therefore the Warburg effect is a favorable catabolic state for all rapidly proliferating mammalian cells with high glucose uptake capacity. Although aerobic glycolysis is less efficient than mitochondrial respiration in terms of ATP yield per glucose uptake, it is more efficient in terms of the required solvent capacity [1].

Our understanding of the amino-acid demand of cancer cells remains incomplete as well. It has been recently shown that the exchange rates of most nutrients correlate poorly with their proliferation rate in 60 tumor-derived cell lines (NCI60) growing in standard culture conditions [12]. These cell lines have been utilized by the National Cancer Institute (NCI) to screen for anticancer drugs [13] and the understanding of their metabolism may aid in the identification of small molecules targeting cancer metabolism. Here we investigate the origin of this apparent inconsistency between metabolite exchange fluxes and cell proliferation, taking into account the variability of cell size and protein content among the NCI60 cell lines. We use these insights to reassess the NCI drug screening data, allowing us to start to personalize drug therapies targeting cancer metabolism.

## Methods

### Cell-doubling times

The doubling times were obtained from the Developmental Therapeutics Program of the NCI (http://dtp.nci.nih.gov/docs/misc/common_files/cell_list.html), and have been confirmed for a subset of cell lines [12].

### Protein synthesis rate estimation

The protein synthesis rate was estimated from the exchange fluxes of essential amino acids, as described in Additional file 1.

### Protein synthesis rate validation

Log-phase cells seeded in 6-well plates the previous day were incubated with pre-warmed RPMI 1640 medium containing 2 μCi/mL (4,5-^3^H)-leucine (Moravek Biochemicals and Radiochemicals, Brea, CA, USA) at 37°C. At predetermined time points (5, 15 and 30 minutes), monolayers were washed twice with ice cold PBS and 0.5 ml of ice cold 10% perchloric acid was added to each well. After 20 minutes incubation, the plates were scraped into a microcentrifuge tube and the samples were centrifuged at 15,000 × g for 10 minutes. The pellet was washed with 10% perchloric acid, centrifuged again and then solubilized with 0.2 M NaOH. The sample was added to a scintillation tube containing 2.5 mL of Ultima-Gold liquid scintillation cocktail (Perkin-Elmer, Waltham, MA, USA), vortexed until the solution was clear and ^3^H counts per minute (CPM) were determined using an LS6000SC Beckman Coulter liquid scintillation counter. At each time point CPMs were normalized by the cell number count: (4,5-^3^H)-leucine incorporation rate was determined as the slope of the plot of CPM/cell as a function of time (Additional file 1: Figure S1).

### Cell size measurements

Cells were grown in RPMI 1640 medium containing 5% FBS and 2 mM L-glutamine at 37°C and 5% CO2, as described previously. Each cell sample was pipetted into the disposable counting chamber and bright-field images were captured for image analysis in duplicate. Cell diameter was measured with the Cellometer Auto T4 (Nexcelom Bioscience LLC, Lawrence, MA, USA). This image cytometer utilizes a bright-field (BR) light microscopy optical setup for image cytometric analysis [14]. The combination of microscope objective (4×) and digital camera provides resolution of approximately 1.05 μm^2^/pixel, which is utilized to calculate accurate cell size of the target sample. The system has a motorized assembly that automatically acquires bright-field images of the target sample. The disposable counting chamber holds precisely 20 μL of the cell sample. Two separate areas are imaged and analyzed on the imaging platform, where the target cells are identified and counted by the Cellometer software. The cell volume was estimated assuming a spherical shape. The validity of the latter assumption is supported by the reported linear relationship between the estimated cell volume and the measured protein content, a surrogate of cell size. The cell size data will be available on the Nexcelom Biosciences website (http://www.nexcelom.com).

### Protein content measurements

NCI60 cell lines were grown in complete medium containing RPMI 1640, with 2 mM L-glutamine and 5% FBS. Cells were seeded in triplicate wells in 6-well plates and maintained at 37°C, 5% CO_2_ until reaching 70 to 80% confluency. Cells were then trypsinized and collected for cell count and total protein extraction. Cell number was determined using the Vi-CELL Cell Viability Analyzer (Beckman Coulter, Indianapolis, IN, USA). The remaining cells from each well were centrifuged at 1500 g for 5 min and washed with 1X PBS. Cell lysates were prepared in radioimmunoprecipitation assay (RIPA) buffer with 1% protease inhibitor cocktail. Protein concentration was determined by Bradford assay (Bio-Rad, Hercules, CA, USA). Protein content/cell was calculated based on total protein content/well and total cell number/well.

### DNA content estimation

The DNA content was estimated from previously reported karyotypes for the NCI60 cell lines [15] and the chromosome sizes reported by Ensembl. DNA content inferred from copy number profiles is in close correspondence with DNA content measured by flow cytometry [16].

### Statistical test for volume dependence

Given a test quantity *Y*_*i*_ (protein content, DNA content or protein synthesis rate) measured across *i* = 1,…,*n* cell lines with cell volumes *V*_*i*_, we assume that:

$$Y_{i}=\mu V_{i}^{\alpha }+\sigma V_{i}^{\beta }X_{i}$$

where *μ* and *σ* are model parameters and α = β = 0 for the volume independent (I) model, α = 1 and β = 0 for the volume dependent mean (VDM), and α = β = 1 for the volume dependent mean and variance (VDMV) model, and *X*_*i*_ are independent random variables with a standard normal distribution. For each model, we assign to μ and *σ* their maximum likelihood estimates (Additional file 1). The validity of each model is then quantified applying the Shapiro-Wilk normality test to:

$$X_{i}=\left(Y_{i}-\mu V_{i}^{\alpha }\right)/\sigma V_{i}^{\beta /2}$$

A model is rejected if the resulting statistical significance falls below 0.05.

### Personalized metabolic models

Personalized metabolic models are described in Additional file 1.

### Gene expression profiles

Affymetrix HG-U133 Plus 2.0 gene expression arrays for the NCI60 cell lines were reported previously [17] and these were downloaded from CellMiner, (http://discover.nci.nih.gov/cellminer/loadDownload.do), GCRMA normalization. Log2 expression values were used for analysis.

### Protein expression profiles

The expression of 194 proteins and phosphoproteins in the NCI60 cell lines was previously reported [18] and these were downloaded from CellMiner, (http://discover.nci.nih.gov/cellminer/loadDownload.do) Log2 protein expression values were used for analysis.

### Gene ontology (GO) analysis

Given the list of genes associated with a GO term, a hypergeometric test was performed to determine the significant enrichment of those genes within the list of genes with at least one Affymetrix HG-U133 Plus 2.0 probe that is positively (negatively) correlated with cell volume.

### Correlation analysis

All reported correlations between metabolic fluxes and cell variables were quantified using the Pearson correlation coefficient (PCC). The statistical significance of the observed PCC was estimated using a permutation test. The statistical significance *P* was computed as the fraction of times the PCC of the permuted variables was as large as, or larger than the observed value in 10^8^ such permutations.

## Results

### The exchange of essential amino acids is proportional to their abundance in the proteome

**Figure 1:**
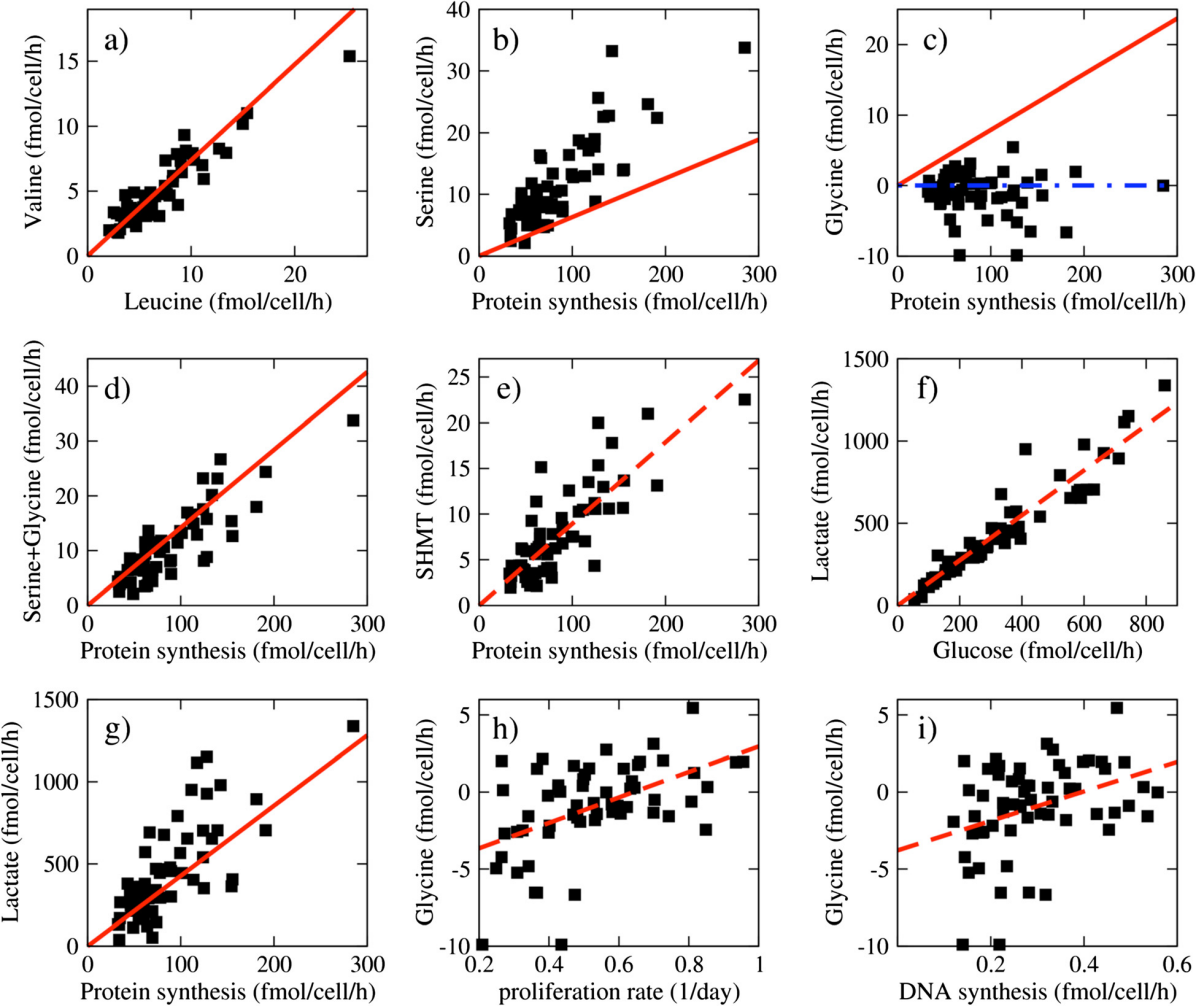
**Import rate of amino acids.** Each square symbol represents a cell line, the red solid lines indicate the expected amount given the demand of protein synthesis and the dashed red lines are linear fits to the data points. **(a)** Valine versus leucine import rate. **(b)** The import rate of serine as a function of the maximum likelihood estimate (MLE) protein synthesis rate. **(c)** The import rate of glycine as a function of the MLE protein synthesis rate. The cell lines below the blue dashed-dotted line export glycine. **(d)** The sum of serine and glycine exchange rates results in a net import that matches the overall serine and glycine requirements for protein synthesis. **(e)** Putative rate of serine to glycine conversion (catalyzed by serine hydroxymethyl transferase), calculated as the expected glycine supply for protein synthesis minus the observed glycine exchange. **(f)** Lactate excretion as a function of the glucose uptake rate. **(g)** Lactate excretion as a function of the MLE protein synthesis rate. The red line represents the ATP demand of protein synthesis (4.27 ATP/amino acid [19]). **(h)** Glycine exchange rate as a function of the proliferation rate. **(i)** Glycine exchange rate as a function of the DNA synthesis rate.

Proteins make up about 70% of cell dry weight. This high protein-content is associated with high metabolic demand for protein synthesis, to balance the basal protein turnover and sustain cell growth [2]. A component of this metabolic demand is the import of essential amino acids (that is, amino acids that cannot be synthesized by human cells) for subsequent protein synthesis. We hypothesized that the import rate of an essential amino acid is proportional to the protein synthesis rate, with a coefficient of proportionality matching its relative abundance in the proteome (Additional file 1: Table S1). The validity of this assumption was tested using the measured metabolic exchange fluxes reported for the NCI60 panel of tumor-derived cell lines [12]. Plotting of the import rate of one essential amino acid versus another produces an evident linear relationship between the two (Figure 1a, symbols). More importantly, the slope matches the ratio of their relative abundance in the human proteome (Figure 1a, red line). Exploiting this relationship, we obtained a maximum likelihood estimate (MLE) of the protein synthesis rate for each cell line in the NCI60 panel. A posteriori, we plotted the import rate of essential amino acids as a function of the MLE protein synthesis rate, corroborating their proportionality (Additional file 1: Figure S1). To validate the MLE protein synthesis rate we quantified the protein synthesis rates of selected cell lines by measuring the rate of (4,5-^3^H)-leucine incorporation into protein. The measurements obtained from both methods are proportional to each other (PCC = 0.99) (Additional file 1: Figure S2).

### The overall exchange of serine and glycine matches the requirements of protein synthesis

Next, we investigated the exchange rate of the non-essential amino acids, serine and glycine, in relation to the estimated protein synthesis rates. Serine was imported from the growth medium in all the reported cancer cell lines, at a magnitude that is proportional but higher than the expected serine demand for protein synthesis (Figure 1b). In contrast, glycine was either imported or exported (that is, secreted into the growth medium) at a magnitude that was proportional, but lower than the expected glycine demand for protein synthesis (Figure 1c). Interestingly, when both contributions are added up, the overall serine + glycine exchange matches what is required for protein synthesis in all NCI60 cell lines (Figure 1d). These data indicate that to a variable extent, in all cancer cells there is a putative net conversion of serine to glycine, catalyzed either by the cytosolic or mitochondrial serine hydroxymethyl transferase (SHMT1 and SHMT2, respectively). Furthermore, the net putative SHMT activity was approximately proportional to the protein synthesis rate (Figure 1e). However, since serine and glycine participate in metabolic pathways other than protein synthesis, we cannot establish a causal link between the protein synthesis rate and the overall exchange rate of serine and glycine.

### The rate of aerobic glycolysis is consistent with the ATP demand of protein synthesis

Protein synthesis is an energy-demanding biosynthetic process. As most cancer cells have a high rate of glycolysis, we first focused on this pathway. As reported previously by Jain *et al.*[12], we also found that a significant fraction of glucose (approximately 70%) was converted to lactate in proportion to the glucose uptake rate (aerobic glycolysis, Figure 1f). Assuming that most of the excreted lactate is formed from glucose and that most of the lactate produced from glucose is excreted, the lactate excretion rate is a surrogate for ATP production from aerobic glycolysis. Surprisingly, the lactate excretion rates were approximately proportional to the protein synthesis rates in a ratio close to the energy demands of protein synthesis (Figure 1g). This scaling relationship indicates that the amount of ATP generated by aerobic glycolysis is approximately equal to the energy requirements for protein synthesis in cancer cells.

The correlation between protein synthesis and aerobic glycolysis rates is supported by previous investigations of protein translation and the mTOR pathway, which plays a major role in its regulation. Treatment with translation initiation inhibitors decreases the glucose uptake and the lactate excretion of cancer cell lines grown *in vitro*[20]. mTORC1 activation increases glucose uptake, whereas treatment with the mTOR inhibitor, rapamycin, decreases glucose uptake [21]. However, further experiments are required to establish a causal link between the energy demands of protein synthesis and the rate of aerobic glycolysis.

### Glycine exchange is correlated with proliferation and DNA synthesis rates

As previously noted by Jain *et al.*[12], we corroborated that the glycine exchange rate is significantly correlated with the proliferation rate of the NCI60 cell lines (PCC = 0.51, *P* = 7 × 10^-6^) (Figure 1h). Furthermore, experiments with ^13^C-labelled glycine demonstrated the incorporation of glycine carbons into purine nucleotides [12]. However, the relationship between glycine exchange and DNA synthesis rates has not been determined. Using the reported karyotypes for the NCI60 cell lines [15], we estimated the DNA content of each cell line. Next we estimated the DNA synthesis rate by multiplying the DNA content by the proliferation rate. We found that the glycine exchange rate was significantly correlated with the DNA synthesis rate (PCC = 0.37, *P* = 0.0026) (Figure 1i).

### The protein synthesis rates are proportional to the cell volumes

**Figure 2:**
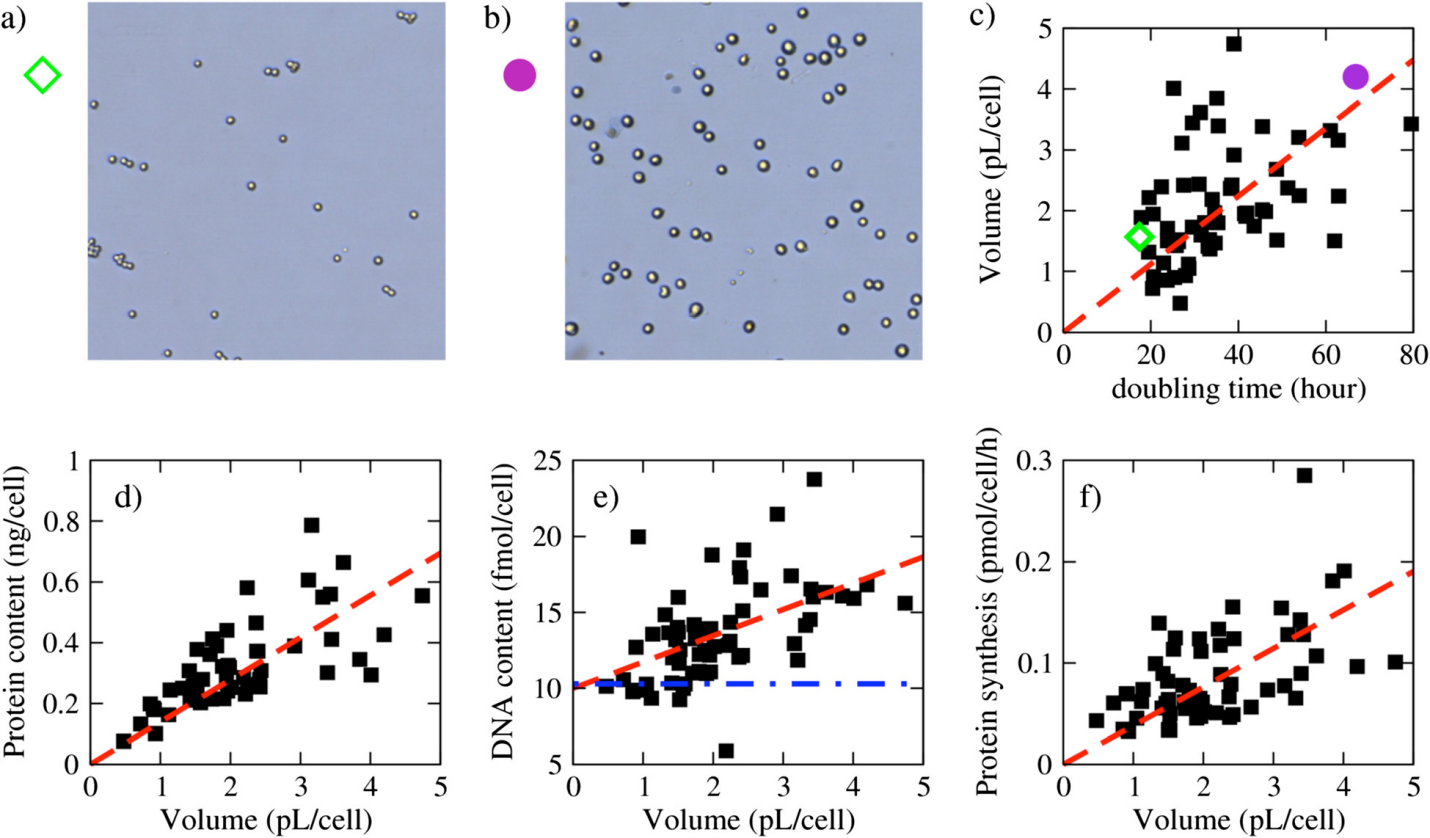
**Correlation between biomass components and doubling time or cell size. (a)** Culture image of the relatively smaller colon cancer cell line HCT-116. **(b)** Culture image of the relatively larger renal cancer cell line A498. **(c)** The cell volume scales in proportion with the doubling time. The diamond and circle represent the HCT-116 and A498 cell lines, respectively. **(d-f)** The protein content, DNA content and protein synthesis rate scale in proportion to the cell volume. The blue dashed-dotted line in panel **(e)** represents the DNA content associated with the diploid genome of a normal cell. All other lines represent linear fits to the data points.

The estimated protein synthesis rates for the NCI60 panel of cancer cell lines were not significantly correlated with their proliferation rate (PCC = 0.088, *P* = 0.25) (Additional file 1: Figure S3). Given that the reported exchange fluxes were reported per cell number, we hypothesized that variations in cell size may be responsible for the lack of correlation. To gain further insight into this issue, we measured cell size and protein content of each cell line in the NCI60 panel, and estimated the cell volume assuming a spherical shape. The estimated cell line volumes are distributed between 1 and 4 pL. Examples of both extremes are shown in Figure 2a and b. There was a positive correlation between cell volumes and the reported doubling times (PCC = 0.45, *P* = 0.00027), indicating that, on average, slowly dividing cells tended to be larger (Figure 2c). Similarly, the protein content per cell was positively correlated with cell doubling time (PCC = 0.38, *P* = 0.0026). However, the estimated DNA content of the NCI60 cell lines did not significantly correlate with their proliferation rate (PCC = 0.17, *P* = 0.092). As anticipated by the correlation of both the protein content and cell volume with the doubling time, we observed positive correlation between the protein content and the cell volume (PCC = 0.69 *P* <10^-6^), with a typical protein concentration of 0.14 g/mL (Figure 2d). The DNA content was also positively correlated with the cell volume (PCC = 0.51, *P* = 0.000032) (Figure 2e) and with the protein synthesis rate (PCC = 0.43, *P* = 0.00078). Finally, the protein synthesis rate per cell was also positively correlated with the cell volume (PCC = 0.55, *P* = 0.000011) (Figure 2f), with a typical rate of 38.1 mmol/L/h.

From Figure 2d we observe that the spread of the protein content around the dashed red line increases with increasing the cell volume. This observation suggests that not only the average but also the variance of the protein content may be a function of the cell volume. The same trend is evident both for the DNA content (Figure 2e) and the protein synthesis rate (Figure 2f). To account for this possibility, we tested three different models representing the dependence of each quantity with the cell volume. The first model assumes that the tested quantity (protein content, DNA content or protein synthesis) is independent of the cell volume, independent (*I*). The second model assumes that the expected value of the tested quantity increases with the cell volume but the variance is independent of the cell volume, volume dependent mean (VDM). The third model assumes that both the expected value and the variance of the tested quantity increase with the cell volume, volume dependent mean and variance (VDMV). For the three quantities we can rule out the independent model (protein content, *p*_*I*_ = 0.0039; DNA content, *p*_*I*_ = 0.0077; protein synthesis, *p*_*I*_ = 0.00028). In the case of the protein content, we could not reject the VDM model (*p*_*VDM*_ = 0.55), neither the VDMV model (*p*_*VDVM*_ = 0.93), although the VDMV seems more likely (*p*_*VDVM*_ = 0.93 vs *p*_*VDM*_ = 0.55). In the case of the DNA content, we can rule out the VDM model (*p*_*VDM*_ = 0.0057) whereas the VDMV model is a good representation of the data (*p*_*VDMV*_ = 0.83). In contrast, for the protein synthesis rate we can rule out the VDMV model (*p*_*VDMV*_ = 0.0069) while the VDM model is a good representation of the data (*p*_*VDM*_ = 0.57). Taken together, these statistical analyses indicate that the average and the standard deviation of the protein and DNA content across cell lines increases proportionally to the cell volumes. The average protein synthesis rate across cell lines also increases with the cell volumes, but with a standard deviation that is independent of the cell volume.

### Association between protein synthesis rates and internal metabolic fluxes

To further understand the impact of cell size and protein synthesis rates on cell metabolism, we developed personalized metabolic models for each cell line in the NCI60 panel, by taking into account their measured cell volume, estimated DNA content and previously reported exchange fluxes. However, we did not constrain the model by the protein content of each cell line. As discussed above, the rate of protein synthesis and the associated protein content can be deduced from the exchange fluxes of essential amino acids. In this way, the comparison of the model-predicted protein content and the measured values may be used as an independent validation. The model-predicted protein synthesis rates are highly correlated with the MLE values (PCC = 0.97, *P* <10^-6^) (Additional file 1: Figure S4a). As theoretically expected, the model predicts slightly lower values. The MLE predicts a protein synthesis rate that is a consensus between the observed essential amino acids import rates. Instead, the metabolic model predicts the protein synthesis rate that is consistent with the limiting essential amino acid, that is, the essential amino acid whose exchange rate results in the lowest protein synthesis rate when assuming that all other essential amino acids can be imported at any rate. The model predicted protein content is also significantly correlated with the measured protein content (PCC = 0.49, *P* = 0.00039) (Additional file 1: Figure S4b). We note that the agreement is not perfect. The differences could be attributed in part to the lack of cell line-specific measurements of the basal protein degradation rate, among other factors. Nevertheless, the model captures the right trend and it can be used to investigate the correlation between internal fluxes and the proliferation or protein synthesis rate.

**Figure 3:**
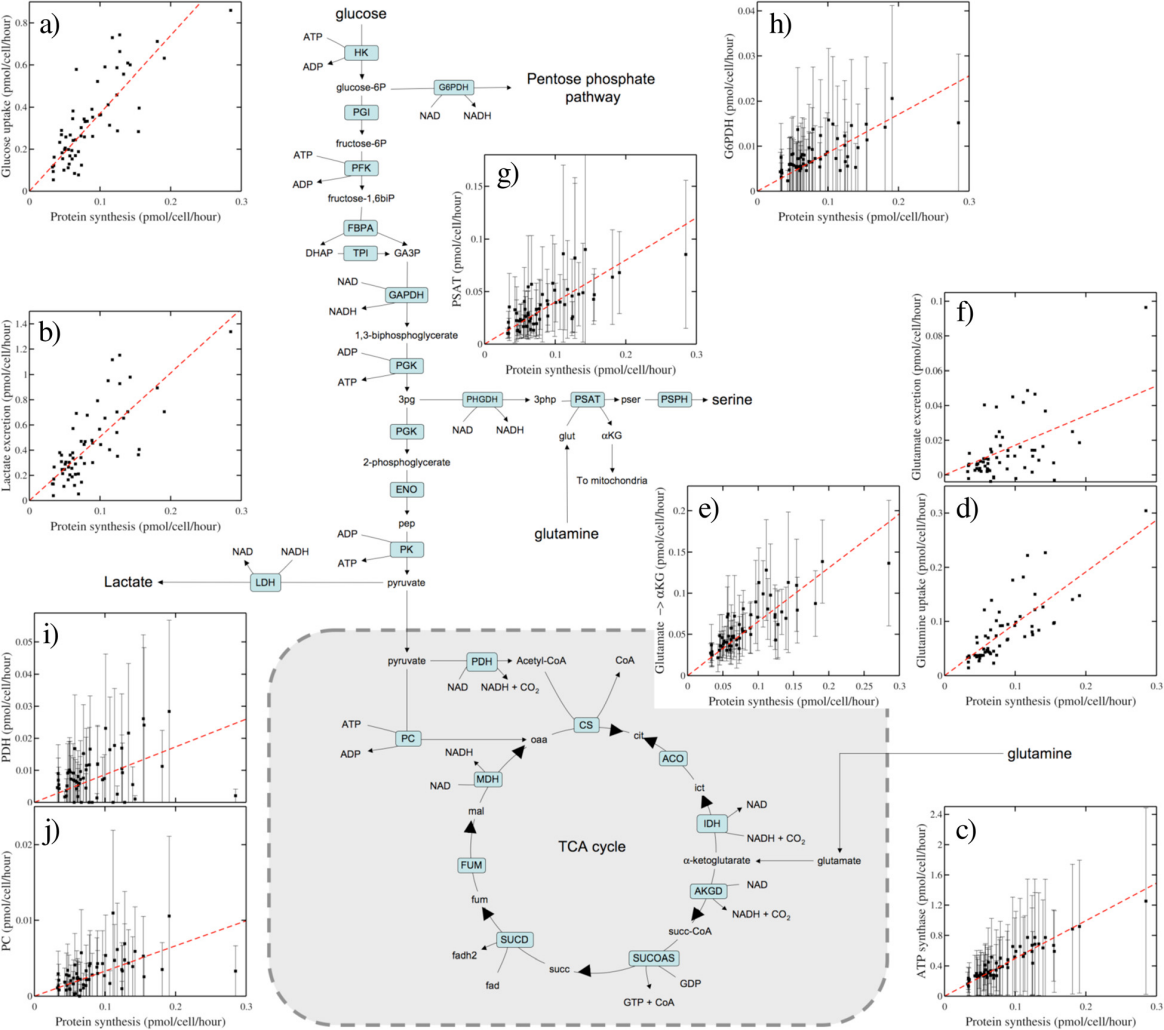
**Metabolic flux distribution as a function of the protein synthesis rate. (a-j)** Selected metabolic pathways are shown. Shown are rates of the reaction indicated in the y-axis as a function of the maximum likelihood estimate (MLE) protein synthesis rate (x-axis) for the NCI60 cell lines. Panels with no error bars represent exchange fluxes that were used as input to the model. Panels with error bars represent flux estimates using our personalized metabolic models. In the latter, each point represents the median over the range of kinetic parameters explored (Additional file 1) and the error bars represent the 90% CI. The dashed lines are linear fits through the origin.

The model-predicted metabolic fluxes can be roughly divided in three major categories based on their magnitude. Glycolysis is in the first category, with rates as high as 1 pmol/cell/h (Figure 3a,b). We also note an ATP synthase catalyzed flux rate in that range (Figure 3c), indicating that OxPhos in the mitochondria contributes to energy generation in an amount comparable to that by glycolysis. Glutaminolysis is in a second category, with intermediate rates around 1/10 pmol/cell/h (Figure 3d). Overall, the imported glutamine is utilized as a precursor amino acid in protein synthesis and converted to glutamate. The produced glutamate is also utilized as a precursor amino acid in protein synthesis, converted to α-ketoglutarate by different transaminases in the cytosol and the mitochondria (Figure 3e), and excreted (Figure 3f). Among the transaminases, phosphoserine transaminase (PSAT) links serine synthesis from 3-phosphoglycerate to glutaminolysis (Figure 3g), as previously reported for breast cancer and melanoma cell lines [22,23]. Finally, the third category comprises reactions with fluxes in the range of 1/100 pmol/cell/h, including the oxidative branch of the pentose phosphate pathway (PPPox, Figure 3h), and the reactions catalyzed by pyruvate dehydrogenase (PDH, Figure 3i) and pyruvate carboxylase (PC, Figure 3j). The rate of all these reactions is significantly correlated with the protein synthesis rate, as can be observed from direct inspection of the panels in Figure 3, and as quantified in Table 1, with the notable exceptions of aspartate and glycine exchange rates.

**Table 1:** Correlation of exchange fluxes with proliferation rate and protein synthesis rate

		**Flux per cell versus proliferation rate**		**Flux per cell versus protein synthesis rate**		**Flux per cell volume versus proliferation rate**	
		**PCC**	***P***	**PCC**	***P***	**PCC**	***P***
	Protein synthesis	0.09	0.250214	1.00	**0.000000**	0.55	**0.000004**
Essential amino acids	Isoleucine	0.05	0.349331	0.96	**0.000000**	0.48	**0.000082**
	Leucine	0.08	0.281351	0.98	**0.000000**	0.54	**0.000008**
	Lysine	0.16	0.108393	0.95	**0.000000**	0.56	**0.000003**
	Methionine	0.20	0.065968	0.92	**0.000000**	0.65	**0.000000**
	Phenylalanine	0.12	0.184280	0.97	**0.000000**	0.59	**0.000001**
	Threonine	−0.01	0.466997	0.92	**0.000000**	0.42	**0.000519**
	Tryptophan	0.01	0.459913	0.83	**0.000000**	0.37	**0.002299**
	Valine	0.06	0.333105	0.97	**0.000000**	0.49	**0.000068**
Non-essential amino acids	Alanine	0.38	**0.000912**	−0.55	**0.000040**	0.11	0.200642
	Arginine	0.10	0.230809	0.45	**0.001921**	0.39	**0.001534**
	Asparagine	0.15	0.134489	0.51	**0.000448**	0.43	**0.000407**
	Aspartate	0.04	0.373865	0.18	0.081690	0.14	0.151826
	Glutamine	−0.23	**0.032499**	0.78	**0.000000**	0.13	0.156961
	Glutamate	0.26	**0.018120**	−0.59	**0.000080**	0.14	0.137636
	Glycine	0.51	**0.000007**	−0.12	0.179629	0.47	**0.000066**
	Proline	0.21	**0.047898**	−0.30	**0.014511**	0.03	0.398548
	Serine	−0.10	0.226831	0.83	**0.000000**	0.29	**0.012954**
	Tyrosine	0.07	0.282791	0.96	**0.000000**	0.52	**0.000014**
Other	Glucose	−0.17	0.092456	0.80	**0.000000**	0.21	0.058257
	Lactate	0.22	**0.043187**	−0.76	**0.000000**	−0.13	0.157716
	PPPox	0.34	**0.004365**	0.66	**0.000000**	1.00	**0.000000**
	PSAT	−0.16	0.104559	0.73	**0.000000**	0.24	**0.033800**
	PDH	0.48	**0.000078**	0.29	**0.018433**	0.72	**0.000000**
	PC	0.02	0.445333	0.52	**0.000207**	0.35	**0.003715**
	Glutamate →α-KG	0.05	0.358935	0.80	**0.000000**	0.64	**0.000000**
	ATP synthase	0.00	0.491256	0.94	**0.000000**	0.52	**0.000014**

Correlations between the exchange fluxes of amino acids, lactate and glucose and the predicted flux of selected reactions, with either the protein synthesis rate per cell, the proliferation rate, or the proliferation rate after normalizing by cell volume. PCC*,* Pearson correlation coefficient; *P*, associated statistical significance; PPPox, oxidative branch of the pentose phosphate pathway; PSAT, phosphoserine transaminase PC, pyruvate carboxylase; α-KG, α-ketoglutarate. Values in bold indicate significance below *P* <0.05.

### Metabolic fluxes correlate with proliferation rate after correcting for cell volume

**Figure 4:**
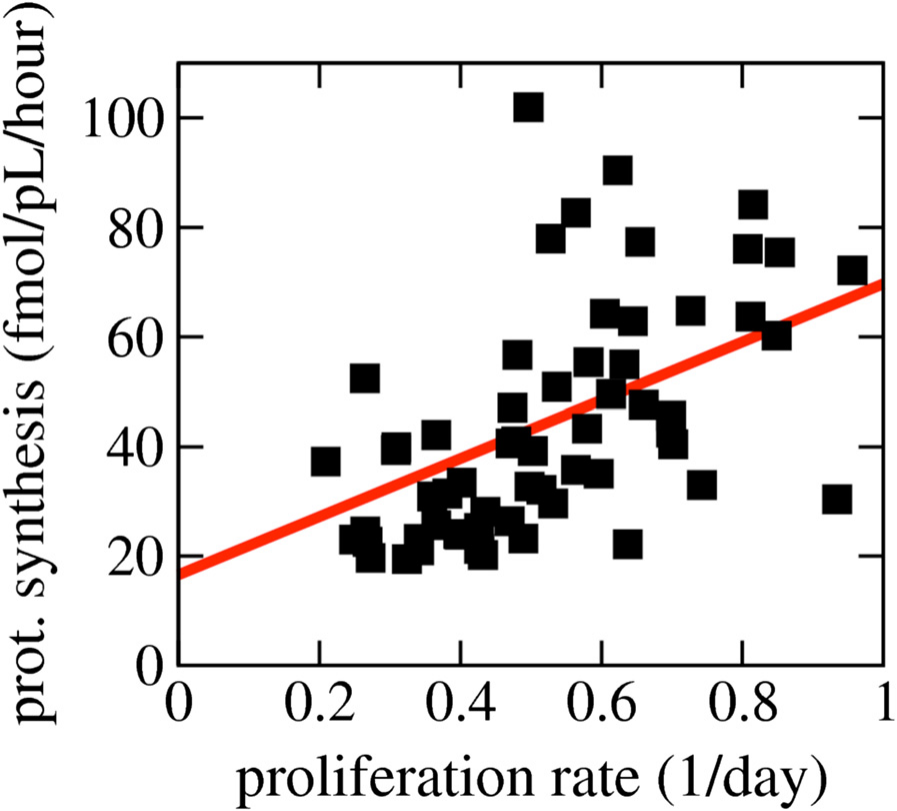
**Correlation between protein synthesis and proliferation rates after normalization by cell volume.** The protein (prot.) synthesis rate per cell volume as a function of the proliferation rate. Each symbol represents a cell line in the NCI60 panel. The red line represents the theoretical expectation (Equation 1).

These analyses may raise the impression that the proliferation rate has no impact on the metabolism of cancer cells. However, after correcting for cell volume and converting the fluxes from per cell to per-cell volume we obtained significant correlation with the proliferation rate. The protein synthesis rate per cell volume was positively correlated with the proliferation rate (PCC = 0.55, *P* = 4 × 10^-6^, Figure 4). Therefore, although larger cells tend to have a higher rate of protein synthesis per cell (Figure 2f), they have a lower protein synthesis rate per cell volume (Figure 4). In contrast, smaller cells tend to have a lower rate of protein synthesis per cell, but a higher protein synthesis rate per cell volume due to their relatively higher proliferation rates (Figure 4).

Theoretically, the protein synthesis rate per cell volume (*f*_*P*_) should be a function of the protein density (*P*_*d*_), the average molecular weight of an amino acid in expressed proteins (*w*_*aa*_), the basal rate of protein turnover (*k*_*D*_) and the proliferation rate (*μ*), following the equation:

(1) $$f_{P}=\frac{P_{d}}{w_{\mathrm{aa}}}\left(k_{D}+\mu \right)$$

As discussed above, the linear scaling of the protein content as a function of the cell volume (Figure 2d) suggest an approximately constant protein density across cell lines of *P*_*d*_ = 0.14 g/mL. *w*_*aa*_ can be estimated by taking into account the average amino acid composition of expressed proteins and the amino acids molecular weight (Additional file 1), obtaining *w*_*aa*_ = 108.89 g/mol. Finally, the basal protein turnover is about *k*_*D*_ = 0.01/h [24]. Using these parameter estimates we can plot the theoretically expected line of the protein synthesis rate per cell volume as a function of the proliferation rate (Equation 1). This theoretical prediction is in very good agreement with the experimental data (Figure 4). If we instead use *k*_*D*_ as a free parameter and fit the theoretical line to the experimental points in Figure 4, we obtain *k*_*D*_ = mean 0.015 ± SD 0.002 protein/h, which is in very good agreement with the previous experimental report of *k*_*D*_ = 0.01/h [24], again supporting the validity of the theoretical line (Equation 1).

Similarly, the exchange flux of all essential amino acids, some non-essential amino acids (arginine, asparagine, glycine, serine, tyrosine) and some of the reported internal fluxes (PPPox, PD, PC, glutamate → αKG, ATP synthase) are also significantly correlated with the proliferation rate when normalized by the cell volume (Table 1). There are some notable exceptions, including the exchange flux of the non-essential amino acids alanine, aspartate, glutamate, glutamine and proline, uptake of glucose (marginally correlated), and lactate excretion (Table 1). Furthermore, as demonstrated previously [12] and above (Figure 1h, Table 1), correlation between glycine and the proliferation rate is evident even without normalizing by the cell size.

### Large cells manifest gene expression patterns of mesenchymal cells

**Figure 5:**
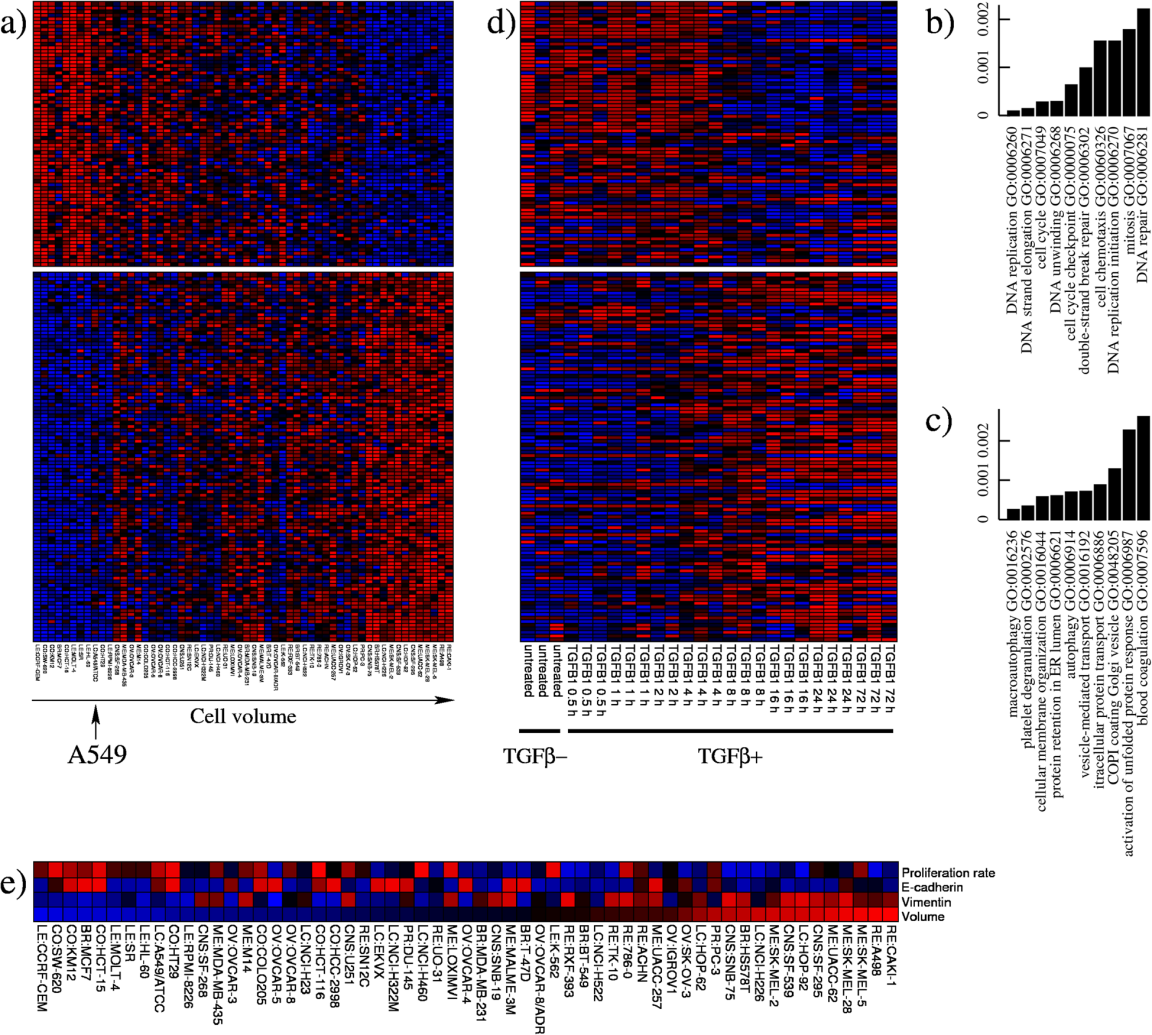
**Gene expression signatures of small/large cells. (a)** Gene expression profile of genes with expression that increased (bottom) or decreased (top) with increasing cell volumes (left to right) across the NCI60 cell lines. **(b)** Gene ontology (GO) terms enriched in genes with expression that decreased from small to large cell lines, quantified by the enrichment significance (y-axis). **(c)** GO terms enriched in genes with expression that increased from small to large cell lines. **(d)** Gene expression profiles of the same genes in the cell line A549 before and after treatment with transforming growth factor (TGF)β. **(e)** Protein expression of vimentin and E-cadherin across the NCI60 cell lines in relation to the cell volume (increases left to right) and the proliferation rate.

To further investigate the differences between small/highly-proliferative cells and large/slowly-proliferating cells we analyzed previously reported basal gene expression profiles for the NCI60 panel of cell lines [17]. We selected genes with expression manifesting high positive correlation with the cell volumes (PCC >0.5) (Additional file 1: Table S2). The expression of these genes clearly increases when going from smaller to larger cell lines (Figure 5a). Similarly, we selected genes with expression manifesting high negative correlation with the cell volumes (PCC < −0.5) (Additional file 1: Table S2). The expression of these genes clearly decreases when going from smaller to larger cell lines (Figure 5a). The positively and negatively correlated gene lists were subjected to GO analysis, to determine the association between annotated pathways and cell volume. The genes with decreased expression in cells with larger cell volume were enriched in GO terms related to DNA replication, cell cycle and DNA repair (Figure 5b), corroborating the negative correlation between cell volume and proliferation rate. In contrast, the genes with increased expression in cells with larger cell volume were enriched in GO terms related to changes in cell morphology, trafficking of proteins between cellular organelles and autophagy (Figure 5c).

Cell morphology remodeling is a characteristic phenotype of mesenchymal cells. We hypothesized that those genes for which expression increases/decreases with increasing cell volume may manifest a similar profile during an epithelial mesenchymal transition (EMT). To test this hypothesis we analyzed previously reported gene expression profiles [25], characterizing the response of the relatively small A549 cell line (indicated by the arrow in Figure 5a) to treatment with transforming growth factor (TGF)β, a canonical inducer of the EMT. The genes with expression that was highly correlated with the cell volume manifested a similar pattern of expression when going from smaller to larger cell lines (Figure 5a) than when treating the A549 cell line with TGFβ (Figure 5d). The set of genes with expression that increased in cells with large cell-volume exhibited increased expression after TGFβ treatment. Similarly, the set of genes with decreasing expression in cells with larger cell volume manifested decreased expression after TGFβ treatment.

If larger cells are characterized by a mesenchymal phenotype then they should express markers of mesenchymal cells. To test this hypothesis we analyzed recently reported reverse-phase protein array quantification of 194 proteins and phosphoproteins in the NCI60 cell lines [18]. The highest positive correlation between protein expression and cell volume was observed for vimentin (PCC = 0.36, *P* = 0.0017) (Additional file 1: Table S3), a standard marker of mesenchymal cells. This significant correlation is visualized in Figure 5c, showing that the protein expression of vimentin is strongly correlated with the cell volume, and both are inversely correlated with the proliferation rate. In contrast, the epithelial marker E-cadherin exhibits the second highest negative correlation between protein expression and cell volume (PCC = −0.20, *P* = 0.062) (Additional file 1: Table S3), which is visually corroborated in Figure 5c. Taken together these data indicate that the larger cells manifest expression signatures of mesenchymal cells.

### Food and Drug Administration (FDA)-approved drugs targeting cells with high protein synthesis or proliferation rate

**Figure 6:**
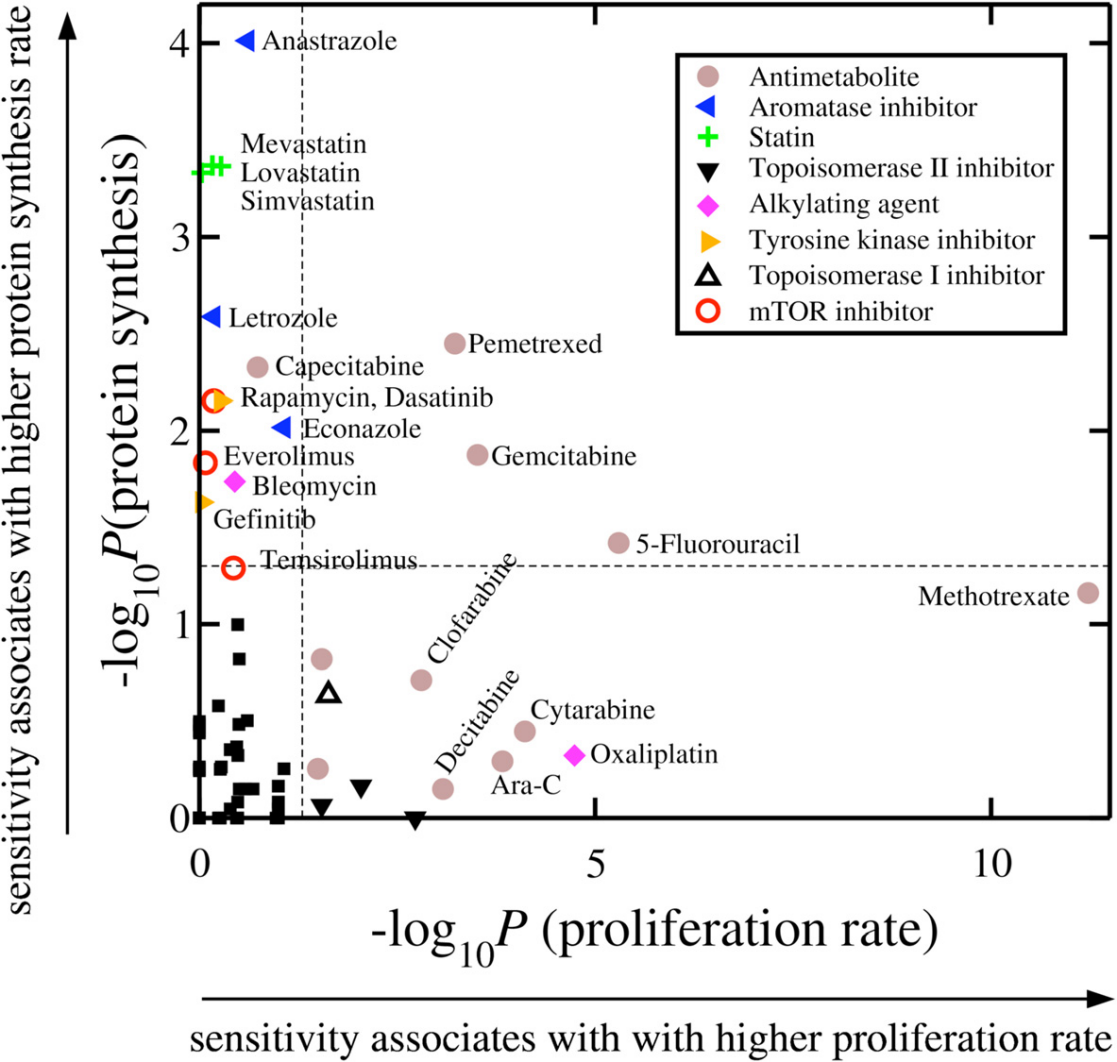
**Implications of cell protein synthesis and proliferation rates for cancer treatment.** Statistical significance is shown for increased *in vitro* sensitivity in cell lines with high protein synthesis rate per cell versus the statistical significance for increased sensitivity in cell lines with high proliferation rate. The horizontal/vertical dashed lines represent the threshold statistical significance of 0.05. Different symbols emphasize different drug classes as indicated in the legend, except for the solid squares that represent other mechanisms not indicated.

These observations indicate that there are metabolically distinct, slowly proliferating large cancer cells with high protein-synthesis rates per cell, and rapidly proliferating small cancer cells with low protein-synthesis rates per cell. We hypothesized that this metabolic difference may have a significant impact on the response to targeted therapies against cancer metabolism. To test this hypothesis, we analyzed *in vitro* data reporting the response of the NCI60 cell lines to 103 FDA-approved drugs [26] (Additional file 1: Table S4). Using our previously established methodology [27], we identified drugs with extremely low IC_50_ values in cells with high proliferation rates relative to those with low proliferation rates, and drugs with extremely low IC_50_ values in cells with high protein-synthesis rates relative to those with low protein-synthesis rates. In agreement with our current knowledge, we found several antimetabolites among the agents that are selective for highly proliferating cells, together with some toposiomerase I/II inhibitors and one alkylating agent (Figure 6). Among the antimetabolites, methotrexate and 5-fluorouracil manifested the highest selectivity (Figure 6). In contrast, we found that aromatase inhibitors, statins and mTOR inhibitors are selectively inhibitory for cells more slowly proliferating with high protein synthesis rates per cell (Figure 6).

## Discussion

Our analyses here indicate that cancer cells grown *in vitro* can be roughly divided into fast proliferating small cells (hyperplastic) with relatively low protein synthesis rates per cell, and slowly proliferating large cells (hypertrophic) with high protein synthesis rates per cell and mesenchymal expression signatures. In turn, the assessment of *in vitro* growth inhibition data provides candidate drugs for the treatment of cancer cells in the hyperplastic and hypertrophic class. As expected, the sensitivity to several antimetabolites correlated with higher proliferation rates, in agreement with previous reports [28,29]. In contrast, high protein synthesis rate is associated with increased sensitivity to mTOR, aromatase, and cholesterol synthesis inhibitors. mTOR is a master regulator of protein synthesis [30] and, therefore, the selectivity of mTOR inhibitors against cancer cells with high protein synthesis rates is not surprising.

Statins and aromatase inhibitors target the cholesterol and estradiol synthesis pathways, respectively, and are not widely considered to have activity against protein synthesis. Statins are currently under intense investigation for their cancer prevention potential [31,32]. The most recent large study, on the entire Danish population, indicates that statin treatment prior to cancer diagnosis is associated with reduced rate of cancer development [31]. The hypothesis for these observations is that the availability of cholesterol may limit the cellular proliferation required for cancer growth. However, the mechanism of action behind this association and whether it holds *in vivo* remains to be determined. In addition to inhibition of cholesterol synthesis by statins, there are reports of statin off-target effects resulting in inhibition of protein synthesis, although a mechanistic understanding of this inhibition is missing [33,34]. From our analysis, we cannot exclude the possibility that larger cancer cells contain more cell membrane and thus require more cholesterol for their proliferation. Activation of mTOR1 increases both protein synthesis and sterol synthesis [21], indicating that these two pathways may be co-regulated. If that were the case, then the association between response to statins and protein synthesis rate could be explained by the correlation between cell volume and protein synthesis rate and a potential correlation between lipid synthesis and cell volume. Therefore, it will be important to investigate whether lipid content and lipid synthesis also correlate with cell volume or surface area in the NCI60 panel.

In the case of aromatase inhibitors we lack a hypothesis for their *in vitro* specificity against large cells with high protein-synthesis rates. Aromatase inhibitors block estrogen synthesis and they are currently used for the treatment of estrogen receptor-positive breast cancer [35]. Further work is required to determine the relevance of this association in the context of other cancer types.

It also remains to be explained why the exchange rate of some amino acids is correlated with the proliferation rate but not with the protein synthesis rate, glycine being the most prominent example. Experiments with ^13^C-labelled glycine demonstrate the incorporation of glycine carbons into purine nucleotides, suggesting a role in DNA synthesis [12]. Here, we have shown that the glycine exchange rate is significantly correlated with the rate of DNA synthesis in the NCI60 panel of cell lines grown *in vitro*. However, the reason why glycine is only imported in highly proliferating cells remains unclear. Indeed, cells could instead increase the serine import and convert serine to glycine, as is the case in slowly proliferating large cells. In general, the switch from one metabolic mode to another takes place when cell metabolism reaches a physico-chemical constraint. A limitation in the serine uptake capacity is unlikely because among cells importing glycine there is a high variability in the uptake of serine. On a different line of reasoning, we note that glycine and the other amino acids showing an atypical behavior (alanine, glutamate, glutamine, and proline) have in common their use as organic osmolytes [36-38]. Thus, the exchange fluxes of these amino acids may be coupled to some mechanism of cell volume regulation. In fact, the glycine exchange rate is also highly correlated with the volume of the NCI60 cell lines (PCC = 0.36, *P* = 0.0029). While at the current stage this is just a hypothesis, it points to a potential relationship between cell volume regulation and molecular crowding in cancer metabolism.

## Conclusions

The NCI60 cell lines display various metabolic activities, and the type of metabolic activity that they possess correlates with their cell volume and protein content. Protein content, DNA content, and protein synthesis rate per cell are proportional to the cell volume. Smaller cells tend to have shorter doubling times. Estimated metabolic fluxes are proportional to the protein synthesis rate and, after correcting for cell volume, to the proliferation rate. Genes overexpressed in smaller cells are enriched for genes involved in cell cycle, while genes overexpressed in large cells are enriched for genes expressed in mesenchymal cells. The later is further corroborated by the induction of those same genes following treatment with TGFβ, and the overexpression of vimentin at the protein level in the larger cells. In addition to cell proliferation, cell volume and/or biomarkers of protein synthesis may predict response to drugs targeting cancer metabolism.

## Abbreviations

CPM: counts per minute; EMT: epithelial mesenchymal transition; FBS: fetal bovine serum; FDA: Food and Drug Administration; GO: gene ontology; MLE: maximum likelihood estimate; NCI: National Cancer Institute; OxPhos: oxidative phosphorylation; PBS: phosphate-buffered saline; PC: pyruvate carboxylase; PCC: Pearson correlation coefficient; PDH: pyruvate dehydrogenase; PPPox: oxidative branch of the pentose phosphate pathway; RIPA: radioimmunoprecipitation assay; RPMI: Roswell Park Memorial Institute; SHMT: serine hydroxymethyl transferase; TGFβ: transforming growth factor β; VDM: volume dependent mean; VDMV: volume dependent mean and variance.

## Competing interests

LLC and JQ declare a financial interest in the image cytometry system used in the manuscript. The authors LLC and JQ are employees of Nexcelom Bioscience, LLC, which is the manufacturer of the Cellometer Auto T4 image cytometer utilized in the manuscript.

## Authors' contributions

AV and ZNO conceived the work. SCD and AV performed the data analysis. AV performed the metabolic modeling. LLC and JQ performed the cell size measurements. SCD and PMT contributed to the experimental characterization of the NCI60 cell lines, under the supervision of KMH and JRB. LLC, JQ, SCD, PMT, JRB, KMH, ZNO and AV wrote the paper. All authors read and approved the final manuscript.

## Supplementary Material

Additional file 1 Maximum likelihood method to estimate the protein synthesis rate, personalized metabolic model of cell metabolism, and supplementary figures and tables. Click here for file

## References

[B1] Vazquez A Liu J Zhou Y Oltvai ZN Catabolic efficiency of aerobic glycolysis: the warburg effect revisited BMC Syst Biol 2010 4 58 10.1186/1752-0509-4-58 20459610 PMC2880972

[B2] Vander Heiden MG Cantley LC Thompson CB Understanding the Warburg effect: the metabolic requirements of cell proliferation Science 2009 324 1029 1033 10.1126/science.1160809 19460998 PMC2849637

[B3] Kozma L Baltensperger K Klarlund J Porras A Santos E Czech MP The ras signaling pathway mimics insulin action on glucose transporter translocation Proc Natl Acad Sci USA 1993 90 4460 4464 10.1073/pnas.90.10.4460 8389451 PMC46531

[B4] Chiaradonna F Sacco E Manzoni R Giorgio M Vanoni M Alberghina L Ras-dependent carbon metabolism and transformation in mouse fibroblasts Oncogene 2006 25 5391 5404 10.1038/sj.onc.1209528 16607279

[B5] Wise DR DeBerardinis RJ Mancuso A Sayed N Zhang XY Pfeiffer HK Nissim I Daikhin E Yudkoff M McMahon SB Thompson CB Myc regulates a transcriptional program that stimulates mitochondrial glutaminolysis and leads to glutamine addiction Proc Natl Acad Sci USA 2008 105 18782 18787 10.1073/pnas.0810199105 19033189 PMC2596212

[B6] Gao P Tchernyshyov I Chang TC Lee YS Kita K Ochi T Zeller KI De Marzo AM Van Eyk JE Mendell JT Dang CV c-Myc suppression of miR-23a/b enhances mitochondrial glutaminase expression and glutamine metabolism Nature 2009 458 762 765 10.1038/nature07823 19219026 PMC2729443

[B7] Matoba S Kang JG Patino WD Wragg A Boehm M Gavrilova O Hurley PJ Bunz F Hwang PM p53 regulates mitochondrial respiration Science 2006 312 1650 1653 10.1126/science.1126863 16728594

[B8] Bensaad K Tsuruta A Selak MA Vidal MN Nakano K Bartrons R Gottlieb E Vousden KH TIGAR, a p53-inducible regulator of glycolysis and apoptosis Cell 2006 126 107 120 10.1016/j.cell.2006.05.036 16839880

[B9] Warburg O On the origin of cancer cells Science 1956 123 309 314 10.1126/science.123.3191.309 13298683

[B10] Beg QK Vazquez A Ernst J de Menezes MA Bar-Joseph Z Barabasi AL Oltvai ZN Intracellular crowding defines the mode and sequence of substrate uptake by Escherichia coli and constrains its metabolic activity Proc Natl Acad Sci U S A 2007 104 12663 12668 10.1073/pnas.0609845104 17652176 PMC1937523

[B11] Vazquez A Beg QK Demenezes MA Ernst J Bar-Joseph Z Barabasi AL Boros LG Oltvai ZN Impact of the solvent capacity constraint on E. coli metabolism BMC Syst Biol 2008 2 7 10.1186/1752-0509-2-7 18215292 PMC2270259

[B12] Jain M Nilsson R Sharma S Madhusudhan N Kitami T Souza AL Kafri R Kirschner MW Clish CB Mootha VK Metabolite profiling identifies a key role for glycine in rapid cancer cell proliferation Science 2012 336 1040 1044 10.1126/science.1218595 22628656 PMC3526189

[B13] Shoemaker RH The NCI60 human tumour cell line anticancer drug screen Nat Rev Cancer 2006 6 813 823 16990858 10.1038/nrc1951

[B14] Chan LL Zhong X Qiu J Li PY Lin B Cellometer vision as an alternative to flow cytometry for cell cycle analysis, mitochondrial potential, and immunophenotyping Cytometry A 2011 79 507 517 21538841 10.1002/cyto.a.21071

[B15] Roschke AV Tonon G Gehlhaus KS McTyre N Bussey KJ Lababidi S Scudiero DA Weinstein JN Kirsch IR Karyotypic complexity of the NCI-60 drug-screening panel Cancer Res 2003 63 8634 8647 14695175

[B16] Popova T Boeva V Manié E Rozenholc Y Barillot E Stern M-H Ltd iP Analysis of Somatic Alterations in Cancer Genome: From SNP Arrays to Next Generation Sequencing Sequence and Genome Analysis I – Humans, Animals and Plants 2013 iConcept Press Ltd

[B17] Pfister TD Reinhold WC Agama K Gupta S Khin SA Kinders RJ Parchment RE Tomaszewski JE Doroshow JH Pommier Y Topoisomerase I levels in the NCI-60 cancer cell line panel determined by validated ELISA and microarray analysis and correlation with indenoisoquinoline sensitivity Mol Cancer Ther 2009 8 1878 1884 10.1158/1535-7163.MCT-09-0016 19584232 PMC2728499

[B18] Federici G Gao X Slawek J Arodz T Shitaye A Wulfkuhle JD De Maria R Liotta LA Petricoin EF 3rd Systems analysis of the NCI-60 cancer cell lines by alignment of protein pathway activation modules with "-OMIC" data fields and therapeutic response signatures Mol Cancer Res 2013 11 676 685 10.1158/1541-7786.MCR-12-0690 23635402

[B19] Duarte NC Becker SA Jamshidi N Thiele I Mo ML Vo TD Srivas R Palsson BO Global reconstruction of the human metabolic network based on genomic and bibliomic data Proc Natl Acad Sci USA 2007 104 1777 1782 10.1073/pnas.0610772104 17267599 PMC1794290

[B20] Santagata S Mendillo ML Tang YC Subramanian A Perley CC Roche SP Wong B Narayan R Kwon H Koeva M Amon A Golub TR Porco JA Jr Whitesell L Lindquist S Tight coordination of protein translation and HSF1 activation supports the anabolic malignant state Science 2013 341 1238303 10.1126/science.1238303 23869022 PMC3959726

[B21] Duvel K Yecies JL Menon S Raman P Lipovsky AI Souza AL Triantafellow E Ma Q Gorski R Cleaver S Vander Heiden MG MacKeigan JP Finan PM Clish CB Murphy LO Manning BD Activation of a metabolic gene regulatory network downstream of mTOR complex 1 Mol Cell 2010 39 171 183 10.1016/j.molcel.2010.06.022 20670887 PMC2946786

[B22] Possemato R Marks KM Shaul YD Pacold ME Kim D Birsoy K Sethumadhavan S Woo HK Jang HG Jha AK Chen WW Barrett FG Stransky N Tsun ZY Cowley GS Barretina J Kalaany NY Hsu PP Ottina K Chan AM Yuan B Garraway LA Root DE Mino-Kenudson M Brachtel EF Driggers EM Sabatini DM Functional genomics reveal that the serine synthesis pathway is essential in breast cancer Nature 2011 476 346 350 10.1038/nature10350 21760589 PMC3353325

[B23] Locasale JW Grassian AR Melman T Lyssiotis CA Mattaini KR Bass AJ Heffron G Metallo CM Muranen T Sharfi H Sasaki AT Anastasiou D Mullarky E Vokes NI Sasaki M Beroukhim R Stephanopoulos G Ligon AH Meyerson M Richardson AL Chin L Wagner G Asara JM Brugge JS Cantley LC Vander Heiden MG Phosphoglycerate dehydrogenase diverts glycolytic flux and contributes to oncogenesis Nat Genet 2011 43 869 874 10.1038/ng.890 21804546 PMC3677549

[B24] Savinell JM Palsson BO Network analysis of intermediary metabolism using linear optimization, I. Development of mathematical formalism J Theor Biol 1992 154 421 454 10.1016/S0022-5193(05)80161-4 1593896

[B25] Sartor MA Mahavisno V Keshamouni VG Cavalcoli J Wright Z Karnovsky A Kuick R Jagadish HV Mirel B Weymouth T Athey B Omenn GS ConceptGen: a gene set enrichment and gene set relation mapping tool Bioinformatics 2010 26 456 463 10.1093/bioinformatics/btp683 20007254 PMC2852214

[B26] Holbeck SL Collins JM Doroshow JH Analysis of food and drug administration-approved anticancer agents in the NCI60 panel of human tumor cell lines Mol Cancer Ther 2010 9 1451 1460 10.1158/1535-7163.MCT-10-0106 20442306 PMC2868078

[B27] Yu X Vazquez A Levine AJ Carpizo DR Allele-specific p53 mutant reactivation Cancer Cell 2012 21 614 625 10.1016/j.ccr.2012.03.042 22624712 PMC3366694

[B28] Schabel FM Jr The use of tumor growth kinetics in planning "curative" chemotherapy of advanced solid tumors Cancer Res 1969 29 2384 2389 5369685

[B29] Munoz-Pinedo C El Mjiyad N Ricci JE Cancer metabolism: current perspectives and future directions Cell Death Dis 2012 3 e248 10.1038/cddis.2011.123 22237205 PMC3270265

[B30] Sabatini DM mTOR and cancer: insights into a complex relationship Nat Rev Cancer 2006 6 729 734 10.1038/nrc1974 16915295

[B31] Nielsen SF Nordestgaard BG Bojesen SE Statin use and reduced cancer-related mortality N Engl J Med 2012 367 1792 1802 10.1056/NEJMoa1201735 23134381

[B32] Bansal D Undela K D'Cruz S Schifano F Statin use and risk of prostate cancer: a meta-analysis of observational studies PLoS One 2012 7 e46691 10.1371/journal.pone.0046691 23049713 PMC3462187

[B33] Rabkin SW Lodha P Kong JY Reduction of protein synthesis and statin-induced cardiomyocyte cell death Cardiovasc Toxicol 2007 7 1 9 10.1007/s12012-007-0003-7 17646677

[B34] Tuckow AP Jefferson SJ Kimball SR Jefferson LS Simvastatin represses protein synthesis in the muscle-derived C(2)C(1)(2) cell line with a concomitant reduction in eukaryotic initiation factor 2B expression Am J Physiol Endocrinol Metab 2011 300 E564 E570 10.1152/ajpendo.00383.2010 21224482 PMC3064004

[B35] Ryan PD Goss PE The evolving role of aromatase inhibitors in the treatment of early-stage breast cancer Nat Clin Pract Oncol 2005 2 596 597 16341095 10.1038/ncponc0360

[B36] Yancey PH Strange K Ompatible and Counteracting Solutes Cellular and Molecular Physiology of Cell Volume Regulation 1994 Boca Raton: CRC Press 400

[B37] Kiehl TR Shen D Khattak SF Jian Li Z Sharfstein ST Observations of cell size dynamics under osmotic stress Cytometry A 2011 79 560 569 21656664 10.1002/cyto.a.21076

[B38] Steeves CL Hammer MA Walker GB Rae D Stewart NA Baltz JM The glycine neurotransmitter transporter GLYT1 is an organic osmolyte transporter regulating cell volume in cleavage-stage embryos Proc Natl Acad Sci USA 2003 100 13982 13987 10.1073/pnas.2334537100 14615585 PMC283532

