# Supplementary material for: The metabolic demands of cancer cells are coupled to their size and protein synthesis rates
Source: Cancer Metab. 2013 Nov 7;1:20. doi: 10.1186/2049-3002-1-20 (PMC4178206; doi:10.1186/2049-3002-1-20)
Supplement: Additional file 1 — Maximum likelihood method to estimate the protein synthesis rate, personalized metabolic model of cell metabolism, and supplementary figures and tables. [file 2049-3002-1-20-S1.pdf]

The metabolic demands of cancer cells are coupled to their size and protein synthesis rates

Sonia C. Dolfi[1], Leo Li-Ying Chan[2], Jean Qiu[2], Philip M. Tedeschi[1], Joseph R. Bertino[1], Kim M. Hirshfield[1], Zoltán N. Oltvai[3], Alexei Vazquez[4,5,*]

[1]Department of Medicine, [4]Department of Radiation Oncology and [5]Center for Systems Biology, Rutgers Cancer Institute of New Jersey, Rutgers, The State University of New Jersey, New Brunswick, USA

[2]Department of Technology R&D, Nexcelom Bioscience LLC, Lawrence, MA, USA

[3]Department of Pathology, University of Pittsburgh School of Medicine, Pittsburgh, PA, USA

Outline

2 Maximum likelihood estimate of the protein synthesis rate

3 Average amino acid molecular weight in the expressed proteome

4 Statistical test for volume dependence

5 Personalized metabolic models

12 Supplementary Figures

16 Supplementary Tables

Maximum likelihood estimate of the protein synthesis rate

The experimentally measured import rates of an essential amino acid are assumed to be proportional to the protein synthesis rate: $f_a = S_a f_P + \Delta f_a$, where S_a is the relative content of amino acid a in proteome (Table S1), f_P is the rate of protein synthesis and Δf_a represents the variability introduced by the experimental procedures. We further assume that the experimental noise Δf_a follows a normal distribution with an amino acid dependent variance σ_a. Under these assumptions the probability to observe the reported exchange rates for essential amino acids is

(Eq. 1) $$P(f \mid f_P, \sigma) = \prod_{a=1}^{n} \frac{1}{\sqrt{2\pi\sigma_a^2}} \exp\left(-\frac{(f_a - S_a f_P)^2}{2\sigma_a^2} \right)$$

where the product is restricted to essential amino acids and n is the number of essential amino acids. Maximizing (Eq. 1) with respect to f_P and σ_a we obtain their maximum likelihood estimates (MLE)

(Eq. 2) $$f_P = \frac{\sum_{a=1}^{n} \frac{S_a f_a}{\sigma_a^2}}{\sum_{a=1}^{n} \frac{S_a^2}{\sigma_a^2}}$$

(Eq. 3) $$\sigma_a^2 = \frac{1}{n} \sum_{a=1}^{n} (f_a - S_a f_P)^2$$

We solve these equations recursively, starting from the assumption that the measurements of all amino acids exchanges have the same variance

(Eq. 4) $$\sigma_a(0) = \sigma$$

(Eq. 5) $$f_P(0) = \frac{\sum_{a=1}^{n} S_a f_a}{\sum_{a=1}^{n} S_a^2}$$

Then, recursively, we compute first $\sigma_a(t)$ using as input $f_P(t\text{-}1)$ and then $f_P(t)$ using as input $\sigma_a(t)$. We continue this procedure until the relative error: $\varepsilon = \text{abs}\left(f_P(t) - f_P(t-1)\right)/f_P(t)$ is smaller than 10^{-6}. The MLE estimates for f_P and σ_a are reported in Tables S5 and S6, respectively.

Average amino acid molecular weight in the expressed proteome

The average amino acid molecular weight in the expressed proteome was calculated as

$$w_{aa} = \sum_a S_a W_a$$

where S_a is the abundance of amino acid a in the expressed proteome and W_a is its molecular weight, as reported in Refs. [9, 21].

Statistical test for volume dependence

Given a test quantity Y_i (protein content, DNA content or protein synthesis rate) measured across i=1,…,n cell lines with cell volumes V_i, we assume that $Y_i = \mu V_i^\alpha + \sigma V_i^\beta X_i$, where μ and σ are model parameters and α=β=0 for the I model, α=1 and β=0 for the VDM model, and α=β=1 for the VDMV model, and X_i are independent random variables with a standard normal distribution. The likelihood to observe the data Y_i given these models is

$$P(Y \mid \mu,\sigma) = \prod_{i=1}^{n} \frac{1}{\sqrt{2\pi\sigma^2 V_i^\beta}} \exp\left(-\frac{\left(Y_i - \mu V_i^\alpha\right)^2}{2\sigma^2 V_i^\beta} \right)$$

For each model, we assign to μ and σ their maximum log-likelihood $L = \ln P(Y \mid \mu,\sigma)$, obtaining

$$\mu_{MLE} = \frac{\sum_{i=1}^{n} Y_i V_i^{\alpha-\beta}}{\sum_{i=1}^{n} V_i^{2\alpha-\beta}}$$

$$\sigma_{MLE}^2 = \frac{1}{n} \sum_{i=1}^{n} \frac{\left(Y_i - \mu_{MLE} V_i^\alpha\right)^2}{V_i^\beta}$$

The validity of each model is then quantified applying a Shapiro-Wilk normality test to

$$X_i = \frac{Y_i - \mu_{MLE} V_i^\alpha}{\sigma_{MLE} V_i^{\beta/2}}$$

A model is rejected if the resulting statistical significance falls below 0.05.

Personalized metabolic models

As starting point, we utilize a genome-scale metabolic reconstruction of a generic human cell [1] that includes most biochemical reactions catalyzed by enzymes encoded in the human genome. We add auxiliary reactions to represent nutrient uptake, excretion of metabolic byproducts, basal ATP demand needed for cell maintenance, basal rate of protein degradation, and synthesis of cell biomass components (proteins, lipids, RNA and DNA) (Vazquez et al [2], Table S1). We assume that the cell is in a steady state where the production and consumption of every metabolite and macromolecules balances, known as the flux balance constraint [1]. We use S_{mi} to denote the stoichiometric coefficient of metabolite m in reaction i. We use F_i to denote the net steady state reaction rate (flux) of the ith reaction per cell, where all reversible reactions are represented by a forward and backward rate, respectively. Reactions are divided into nutrient import reactions (I), reactions taking place in the cytosol (C) and reactions taking place in the mitochondria (M). We use ϕ_c to denote the relative cell volume fraction occupied by the cth cellular compartment. In particular, we denote by N_0 the non-metabolic protein content per cell and by ϕ_0 its occupied volume fraction. We assume the proliferation rate (μ) and the relative volume fraction occupied by macromolecules and organelles (ϕ_{max}) are known and are given as input parameters of the model. Finally, we estimate the metabolic fluxes and compartment densities as the solution of the following optimization problem:

Find the F_i and ϕ_c that maximize the total content of non-metabolic protein

(1) $$N_0$$

subject to the metabolic constraints: flux balance constraints

(2) $$\sum_i S_{mi} F_i = 0$$

minimum/maximum flux constraints

(3) $$v_{i,\min} \le F_i \le v_{i,\max}$$

minimum/maximum volume fraction constraints

(4) $$0 \le \phi_c \le \phi_{\max}$$

molecular crowding constraints

(5) $$\begin{aligned} &\sum_{i\in C} a_i F_i \le \phi_C V \\ &\sum_{i\in M} a_i F_i \le \phi_M V \\ a_{M,ATP} &\sum_{i\in M | S_{ATP,i}>0} S_{ATP,i} F_i \le \phi_M V \\ &\phi_0 + \phi_C + \phi_M \le \phi_{\max} \end{aligned}$$

where V is the cell volume, c_i is the nutrient import cost associated with the uptake reaction i, $a_i=v_i/k_{eff,i}$ are the crowding coefficients of metabolic enzymes (enzyme molar volume / enzyme effective turnover)[3], and $a_{M,ATP}=v_{s,M}/r_M$ the crowding coefficient of mitochondria ATP generation (ATP synthesis rate per mitochondria mass / mitochondria specific volume) [4, 5]. As a difference with our previous work [2], here we express the metabolic fluxes F_i in units of mol/cell/hour. We can recover our previous equations recalling that the metabolic flux per cell volume is given by $f_i=F_i/V$.

Personalized models

The model is tailored for each cell line by specifying the proliferation rate μ, the cell volume V, the DNA conent and the reported exchange fluxes. The model reaction fluxes representing exchange fluxes are fixed to the experimentally measure values, by setting $v_{i,min}=v_{i,max}=F_{i,reported}$.

Proliferation rate

The doubling time t_D of each cell line in the NCI60 panel was obtained from the Developmental Therapeutics Program at the NCI (http://dtp.nci.nih.gov/docs/misc/common_files/cell_list.html). Proliferation rates were computed as $\mu=\ln(2)/t_D$.

Nuclear DNA content

The nuclear DNA content of each cell line in the NCI60 panel was estimated using the reported karyotypes for these cell lines [6] and the chromosome lengths reported by Ensembl. Specifically, using the reported copy number for each chromosome band we calculated an average karyotype for each chromosome. Multiplying the average chromosome karyotype by the chromosome length in base pairs and summing over all chromosomes we obtained the DNA content in base pairs (nucleotides/cell). The latter was coverted to mol/cell after dividing by the Avogadro number.

The nuclear DNA content of a normal cell was calculated similarly, using as input the normal chromosomes copy number.

Mitochondrial DNA

The mitochondrial DNA (mtDNA) was assumed proportional to the mitochondrial volume, with a density of 0.3 fmol of mtDNA/pL. The density was calculated based on the reported mitochondrial DNA (~0.75 μg/10^6 cells) and protein (~2.5 mg/10^6 cells) content in chick embryo fibroblasts (Table III in Ref. [7]). Dividing the mtDNA mass per cell by the average DNA molecular weight (~331 g/mol) we obtain a density of 2.3 fmol mtDNA/cell, and dividing by the mitochondrial protein content a density of 0.92 μmol mtDNA/g of mitochondrial protein. Multiplying the latter number by the specific volume of mitochondria (2.6 mL/g protein [8]), we obtain a density of 0.3 fmol mtDNA/pL of mitochondria.

RNA

The RNA composition was estimated by their relative abundance per cell dry weight [9]. The abundance per cell dry weight were converted to abundance per cell volume after dividing by the typical cell specific volume 4.3 mL/g [10]. The abundance per cell volume was finally converted to abundance per cell by multiplying by our measurements of cell volume for each of the cell lines on the NCI60 panel.

Lipids

The lipids composition was estimated by their relative abundance per cell dry weight [9]. The abundance per cell dry weight were converted to abundance per cell volume after dividing by the typical cell specific volume 4.3 mL/g [10]. The abundance per cell volume was finally converted to abundance per cell by multiplying by our measurements of cell volume for each of the cell lines on the NCI60 panel.

Protein content

Proteins are divided into cytosolic and mitochondrial proteins. Within the cytosol, proteins were divided into three pools: ribosomal-, components of metabolic enzyme complexes-, and non-metabolic proteins. Each ribosome contributes to n_{PR}=82 proteins/ribosome (49 in the 60S and 33 in the 40S subunits [11]). The ribosome related protein concentration was computed as $P_{CR}=n_{PR}f_{CP}/k_R$, where f_{CP} is the cytosolic protein synthesis rate and k_R the protein synthesis rate per ribosome. Each enzyme contributes with n_{PE}=2.4 proteins in average, estimated as median enzyme molecular weight (98,750 g/mol, reported above) divided by the median molecular weight of a human protein (40,835 g/mol). The median molecular weight of a human protein was estimated from the median protein length (355 amino acids [12]) and the typical amino acid composition [9]. The enzyme related cytosolic protein concentration was computed as $P_{CE}=\Sigma_i n_{PE}f_i/k_i$. A similar subdivision is carried on for mitochondrial

proteins.

Kinetic parameters

The effective turnover numbers, $k_{eff,i}$, quantify the reaction rate per enzyme molecule. For example, for an irreversible single substrate reaction satisfying Michaelis-Menten kinetics, $k_{eff}=kS/(K+S)$, where k is the enzyme turnover number, K the half-saturation concentration and S the substrate concentration. The turnover numbers of some human enzymes are reported in the BRENDA database [13]. They have a typical value of 10 sec^{-1} and a significant variation from 1 to 100 sec^{-1} (Vazquez et al [2], Table S2). However, for most reactions we do not know the turnover number, the kinetic model, or the metabolite concentrations, impeding us to estimate k_{eff}. To cope with this indeterminacy we performed a sampling strategy, whereby the $k_{eff,i}$ were sampled from a reasonable range of values, and then focused on median and the 90% confidence intervals (see Sensitivity analysis below).

Crowding coefficients

Dividing the mitochondrium specific volume (3.15 mL/g in mammalian liver [14] and 2.6 mL/g in muscle [8]) by the rate of ATP production per mitochondrial mass (0.1-1.0 mmol ATP/min/g [15-17]) we obtain a_M values between 0.0026 to 0.032 min/mM. Except when specified, we use the median 0.017 min/mM. Dividing the ribosome molar volume (v_R = 4,000 $nm^3 \times 6.02\ 10^{23}$/mol=2.4 L/mmol) by the rate of protein synthesis per ribosome (0.67 proteins/min [18]) we obtain a_R=3.6 min/mM. The enzyme crowding coefficients were estimated as $a_i=v_E/k_i$. Multiplying the median molecular weight of human enzymes (98,750 g/mol, Vazquez et al [2], Table S2) by the enzymes specific volume (approximated by the specific volume of spherical proteins, 0.79 mL/g [19]) we obtain an estimated enzymes molar volume of v_E=0.078 L/mmol.

Maximum macromolecular density

The maximum macromolecular density cannot exceed 100% of the cell volume, therefore ϕ_{max}=1. We have previously used the upper bound of 0.4 based on the typical macromolecular density of cells. However, there may be some variability among cancer cell lines. Furthermore, the constraint over the exchange fluxes of essential amino acids determines the protein content, which is the major component of the cell biomass.

Protein synthesis

The flux balance equation for proteins synthesized in the cytosol is formulated as follows. We account for three major categories, proteins not associated with metabolism, proteins that are components of

enzyme complexes and that are encoded in nuclear genes, and the cytosol ribosomal proteins, with their concentrations (moles/cell volume) denoted by P_0, P_{NE}, and P_{CR}, respectively. In proliferating cells, these concentrations will decrease at a rate $(\mu+k_D)(P_0+P_{NE}+P_{CR})$, where μ represents the contribution to cell growth and k_D=0.01/h [20] is the basal protein turnover rate. Putting all these elements together, the balance between protein turnover and synthesis implies f_{CP}-$k_D[P_0+ n_{PE}\Sigma_i(f_i/k_{eff,i}) + n_{PR}\, f_{CP}/k_R]=0$, where the index i runs over all enzyme catalyzed reactions excluding ATP synthase, NADH dehydroganase, complex III, and complex IV. In a similar manner we formulated the flux balance equation for mitochondrial proteins encoded in the mitochondrial DNA. The mitochondrial protein synthesis reaction accounted for the synthesis of ATP synthase, NADH dehydroganase, complex III, complex IV and the mitochondria ribosomes.

DNA synthesis

We explicitly considered a nuclear and a mitochondrial DNA synthesis reaction, each matching the DNA synthesis demands of their respective compartment upon cell proliferation.

Flux bounds

All reversible reactions were represented by an irreversible reaction on each direction with their own effective turnover number $k_{eff,i}$. Flux bounds were set to $v_{i,min}$=0 and $v_{i,max}=\infty$, except for reported exchange fluxes ($v_{i,min}=v_{i,max}=F_{i,reported}$) and for the auxiliary reactions representing the basal rate of protein turn over ($v_{i,min}=v_{i,max}=k_D$) and the biomass production reaction ($v_{i,min}=v_{i,max}=\mu$).

Simulations

The optimization problem in equations (1)-(5) was solved in Matlab, using the linear programming function `linprog`.

Sensitivity analysis

The turnover numbers of human enzymes k have significant variations from 1 to 100 sec^{-1} and the distribution of $\log_{10}(k)$ is approximately uniform in this range (Vazquez et al [2], Table S2). Based on this data we sampled the $\log_{10}(k_{eff})$ values from a uniform distribution in the range between $\log_{10}(1)$ to $\log_{10}(100)$. For each specified condtion we run 100 simulations. On each simulation, for each reaction, a value of $k_{eff,i}$ is extracted from the distribution described above. With this set of $k_{eff,i}$ parameters we then solve the optimization problem (1)-(5) and obtain estimates for the reaction rates. Based on the 100 simulations we finally estimate the median and 90% confident intervals for the rate of each reaction. 100 simulations were proven to be sufficient to capture the overall range of behavior, since

running 1,000 simulations did not result in a significant change in the median and 90% confidence intervals.

References

1. Mo ML, Jamshidi N, Palsson BO: **A genome-scale, constraint-based approach to systems biology of human metabolism**. *Mol Biosyst* 2007, **3**(9):598-603.
2. Vazquez A, Markert EK, Oltvai ZN: **Serine biosynthesis with one carbon catabolism and the glycine cleavage system represents a novel pathway for ATP generation**. *Plos One* 2011, **6**(11):e25881.
3. Vazquez A, de Menezes MA, Barabasi AL, Oltvai ZN: **Impact of limited solvent capacity on metabolic rate, enzyme activities, and metabolite concentrations of S. cerevisiae glycolysis**. *PLoS Comput Biol* 2008, **4**(10):e1000195.
4. Vazquez A, Liu J, Zhou Y, Oltvai ZN: **Catabolic efficiency of aerobic glycolysis: The Warburg effect revisited**. *BMC Syst Biol* 2010, **4**:58.
5. Vazquez A, Oltvai ZN: **Molecular crowding defines a common origin for the warburg effect in proliferating cells and the lactate threshold in muscle physiology**. *Plos One* 2011, **6**(4):e19538.
6. Roschke AV, Tonon G, Gehlhaus KS, McTyre N, Bussey KJ, Lababidi S, Scudiero DA, Weinstein JN, Kirsch IR: **Karyotypic complexity of the NCI-60 drug-screening panel**. *Cancer Res* 2003, **63**(24):8634-8647.
7. D'Agostino MA, Nass MM: **Specific changes in the synthesis of mitochondrial DNA in chick embryo fibroblasts transformed by Rous sarcoma viruses**. *J Cell Biol* 1976, **71**(3):781-794.
8. Schwerzmann K, Hoppeler H, Kayar SR, Weibel ER: **Oxidative capacity of muscle and mitochondria: correlation of physiological, biochemical, and morphometric characteristics**. *Proc Natl Acad Sci U S A* 1989, **86**(5):1583-1587.
9. Sheikh K, Forster J, Nielsen LK: **Modeling hybridoma cell metabolism using a generic genome-scale metabolic model of Mus musculus**. *Biotechnol Prog* 2005, **21**(1):112-121.
10. Frame KK, Hu WS: **Cell volume measurement as an estimation of mammalian cell biomass**. *Biotechnol Bioeng* 1990, **36**(2):191-197.
11. Alberts B: **Molecular biology of the cell**, 5th edition. edn. New York, N.Y.,: Garland Science; 2008.
12. Brocchieri L, Karlin S: **Protein length in eukaryotic and prokaryotic proteomes**. *Nucleic Acids Res* 2005, **33**(10):3390-3400.
13. Schomburg I, Chang A, Schomburg D: **BRENDA, enzyme data and metabolic information**. *Nucleic Acids Res* 2002, **30**(1):47-49.
14. Glas U, Bahr GF: **Quantitative study of mitochondria in rat liver. Dry mass, wet mass, volume, and concentration of solids**. *J Cell Biol* 1966, **29**(3):507-523.
15. Wibom R, Hultman E, Johansson M, Matherei K, Constantin-Teodosiu D, Schantz PG: **Adaptation of mitochondrial ATP production in human skeletal muscle to endurance training and detraining**. *J Appl Physiol* 1992, **73**(5):2004-2010.
16. Short KR, Nygren J, Barazzoni R, Levine J, Nair KS: **T(3) increases mitochondrial ATP production in oxidative muscle despite increased expression of UCP2 and -3**. *Am J Physiol Endocrinol Metab* 2001, **280**(5):E761-769.
17. Hou XY, Green S, Askew CD, Barker G, Green A, Walker PJ: **Skeletal muscle mitochondrial ATP production rate and walking performance in peripheral arterial disease**. *Clin Physiol*

Funct Imaging 2002, **22**(3):226-232.

18. Princiotta MF, Finzi D, Qian SB, Gibbs J, Schuchmann S, Buttgereit F, Bennink JR, Yewdell JW: **Quantitating protein synthesis, degradation, and endogenous antigen processing**. *Immunity* 2003, **18**(3):343-354.
19. Lee B: **Calculation of volume fluctuation for globular protein models**. *Proc Natl Acad Sci U S A* 1983, **80**(2):622-626.
20. Savinell JM, Palsson BO: **Network analysis of intermediary metabolism using linear optimization. I. Development of mathematical formalism**. *J Theor Biol* 1992, **154**(4):421-454.
21. Duarte NC, Becker SA, Jamshidi N, Thiele I, Mo ML, Vo TD, Srivas R, Palsson BO: **Global reconstruction of the human metabolic network based on genomic and bibliomic data**. *Proc Natl Acad Sci U S A* 2007, **104**(6):1777-1782.
22. Holbeck SL, Collins JM, Doroshow JH: **Analysis of Food and Drug Administration-approved anticancer agents in the NCI60 panel of human tumor cell lines**. *Mol Cancer Ther* 2010, **9**(5):1451-1460.

Supplementary Figures

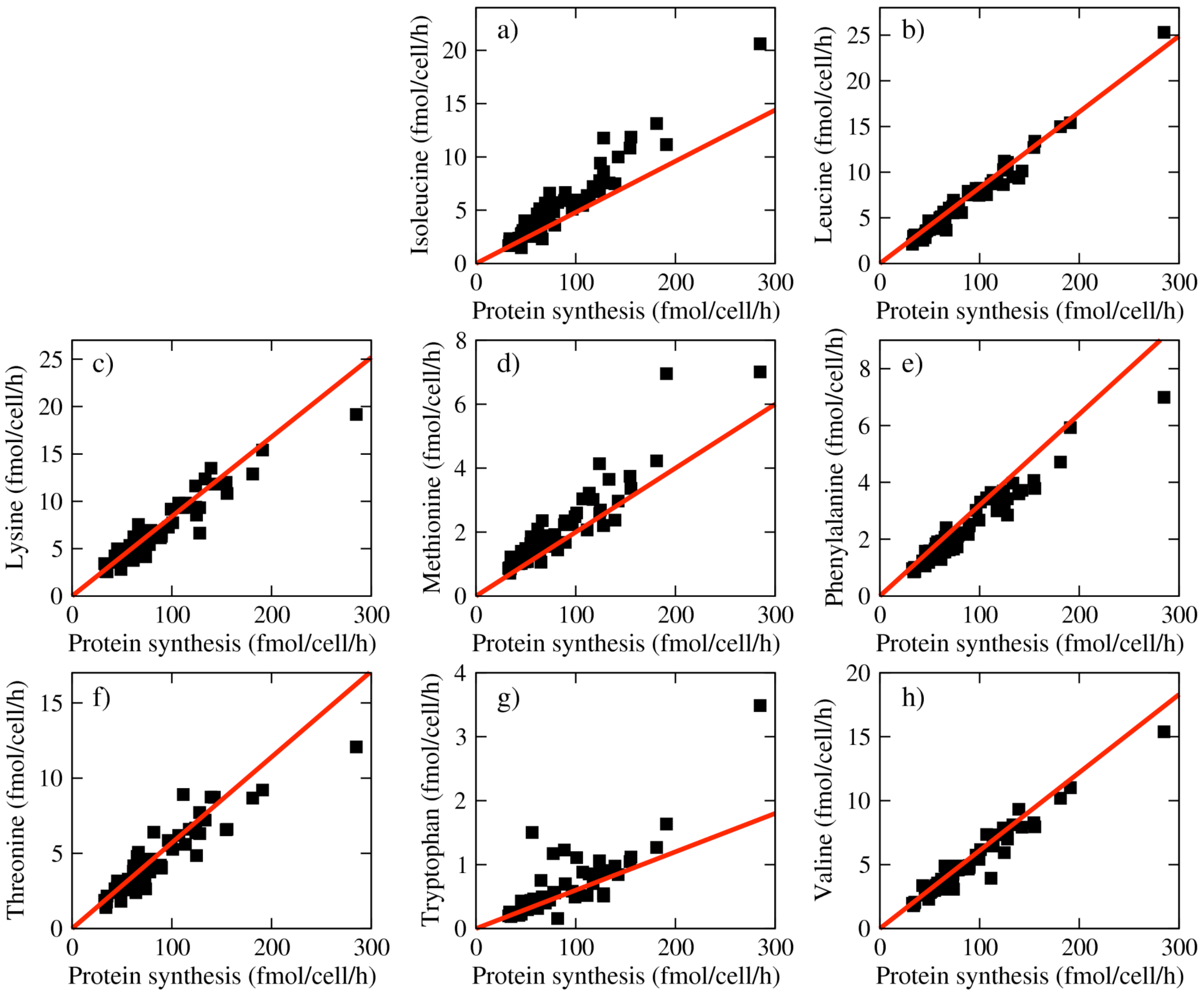

Figure S1: Import rate of essential amino acids as a function of the MLE protein synthesis rate. a-h) Each square symbol represents a cell line from the NCI60 panel and the red solid line represents the expected ratio as determined from the relative amino acid abundance.

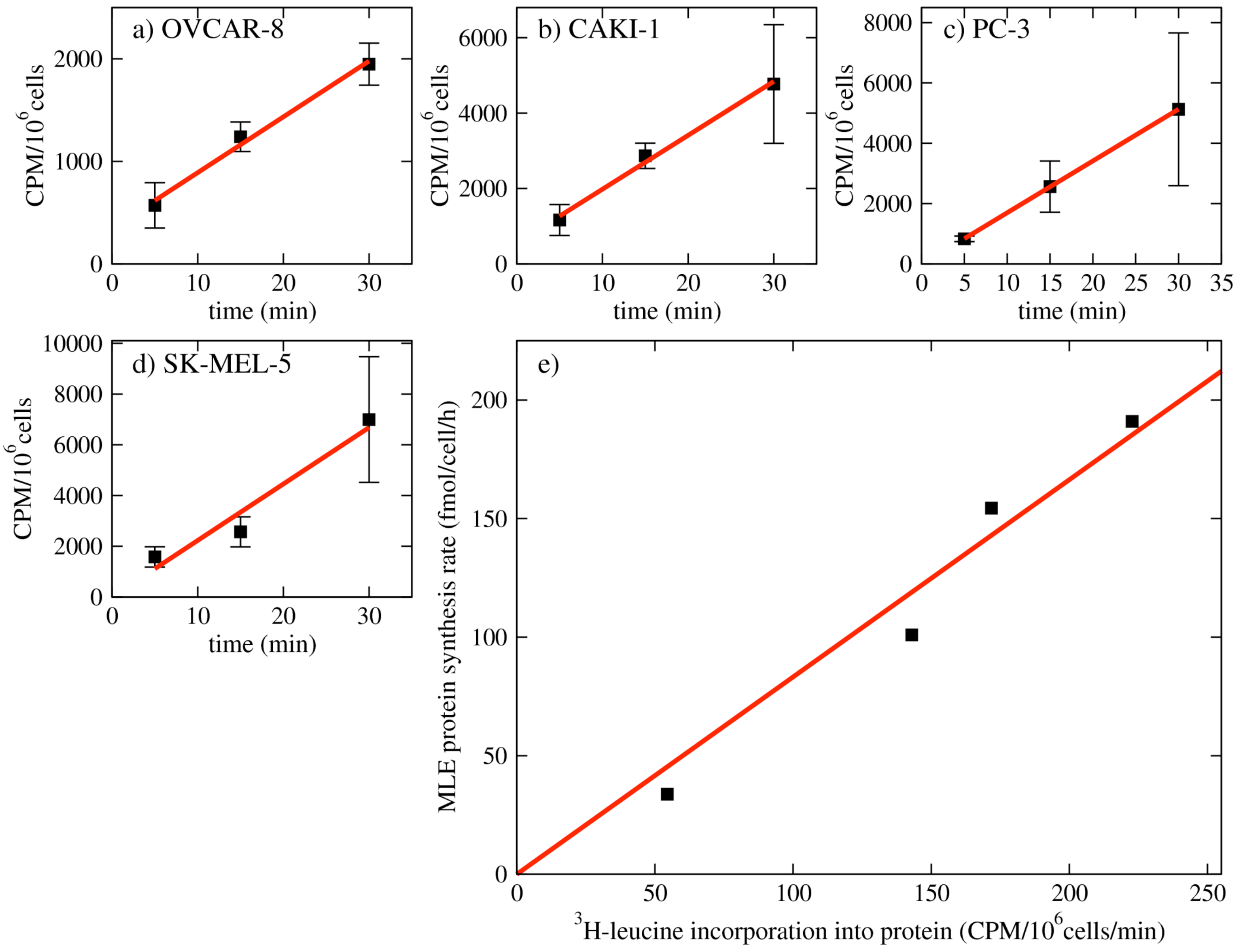

Figure S2: Validation of the MLE protein synthesis rates. a-d) [4,5-^{3}H]-leucine incorporation into protein as a function of time for four selected cell lines. The red lines are linear fits to the data points. e) The MLE protein synthesis rate as a function of the [4,5-^{3}H]-leucine incorporation rate into protein. Each data point represents a cancer cell line and the solid line represents a linear fit intercepting the origin.

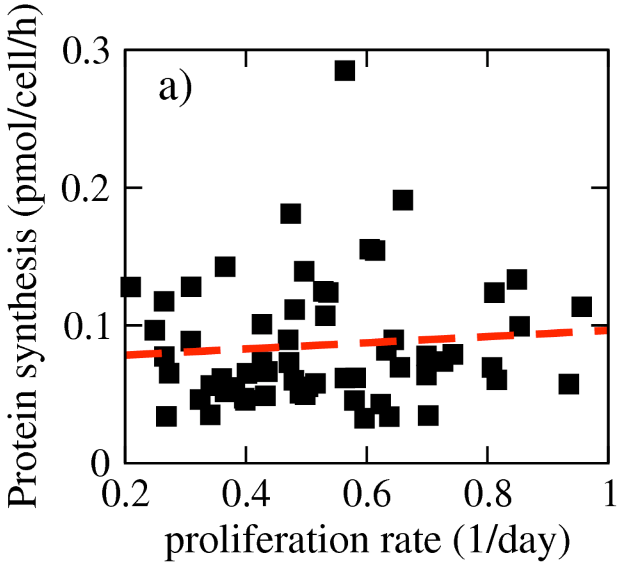

Figure S3: Protein synthesis rate per cell as a function of the proliferation rate. Each square symbol represents a cell line from the NCI60 panel and the dashed red line is a linear fit to the data points**.**

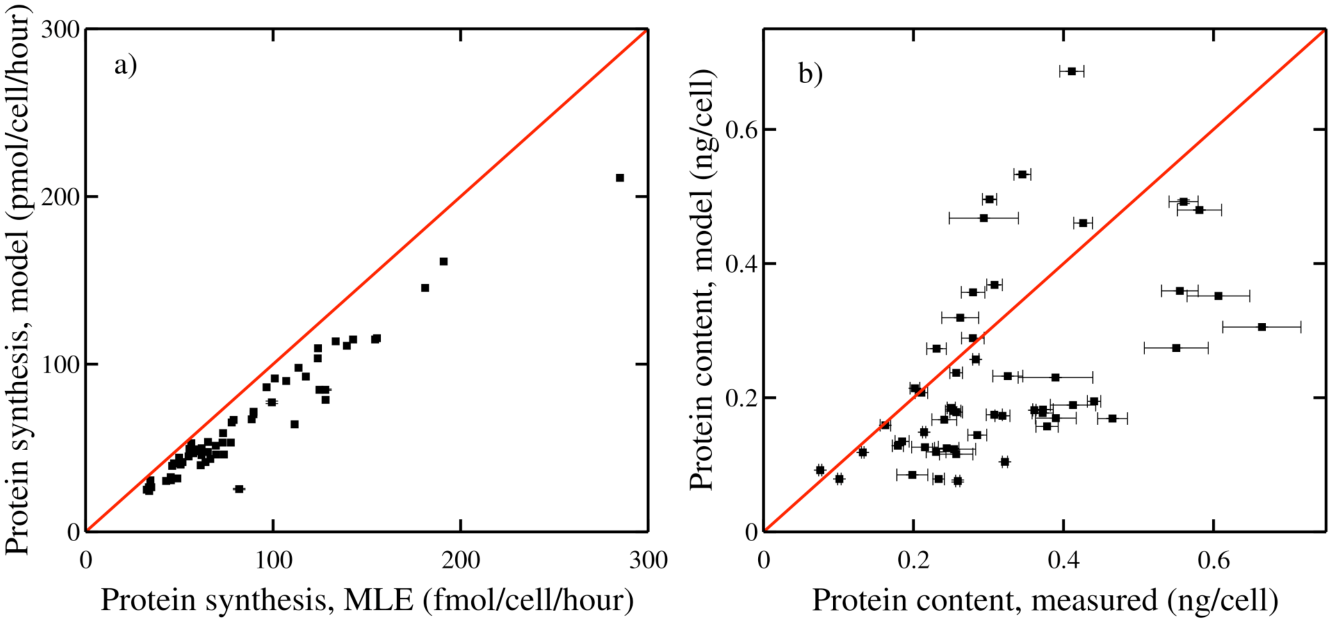

Figure S4: Correlation of measured and estimated protein contents. a) Scatter plot of the model predicted protein synthesis rate vs the MLE protein synthesis rate. b) Scatter plot of the model predicted protein content vs the measured protein content. Each point represents a cell line and the diagonal line represents the case when both values are equal. Although they are not visible due to their small size, there are vertical error bars representing the 90% confidence interval due to variations in the model kinetic parameters. The horizontal error bars in panel b) represent the standard deviation of the protein content measurements.

Supplementary Tables

Amino acid	S_a (mol/mol)
histidine	0.021
isoleucine	0.048
leucine	0.083
lysine	0.084
methionine	0.020
phenylalanine	0.032
threonine	0.057
tryptophan	0.006
valine	0.061
alanine	0.088
arginine	0.055
asparagine	0.042
aspartate	0.053
cysteine	0.021
glutamate	0.057
glutamine	0.047
glycine	0.079
proline	0.046
serine	0.063
tyrosine	0.027

Table S1: Relative abundance of essential and non-essential amino acids in the expressed proteome [9, 21].

Table S2: Genes whose expression correlates with cell volume. The list of Affymetrix HG-U133 Plus 2.0 array probes whose expression values were highly correlated with the cell volumes (*PCC*>0.5) or highly negatively correlated with the cell volume (*PCC*<-0.5) in the NCI60 cell lines.

Positively correlated					Negatively correlated			
Probe	Gene	*PCC*	*p*		Probe	Gene	*PCC*	*p*
200757_s_at	CALU	0.65	0.00E+00		200773_x_at	PTMA	-0.68	0.00E+00
200756_x_at	CALU	0.63	0.00E+00		219067_s_at	NSMCE4A	-0.66	0.00E+00
201828_x_at	FAM127A	0.61	0.00E+00		216515_x_at	PTMA	-0.66	1.00E-06
200755_s_at	CALU	0.60	0.00E+00		202521_at	CTCF	-0.63	0.00E+00
214845_s_at	CALU	0.60	0.00E+00		209753_s_at	TMPO	-0.62	0.00E+00
202733_at	P4HA2	0.60	0.00E+00		208549_x_at	LOC441454	-0.61	0.00E+00
200665_s_at	SPARC	0.59	0.00E+00		224944_at	TMPO	-0.61	1.00E-06
209578_s_at	POFUT2	0.59	0.00E+00		211376_s_at	NSMCE4A	-0.60	0.00E+00
225303_at	KIRREL	0.59	0.00E+00		203388_at	ARRB2	-0.60	1.00E-06
212509_s_at	MXRA7	0.59	0.00E+00		209754_s_at	TMPO	-0.59	4.00E-06
206050_s_at	RNH1	0.58	3.00E-06		201038_s_at	ANP32A	-0.59	0.00E+00
213455_at	FAM114A1	0.58	3.00E-06		203276_at	LMNB1	-0.59	1.00E-05
226697_at	FAM114A1	0.58	0.00E+00		218911_at	YEATS4	-0.57	5.00E-06
202133_at	WWTR1	0.57	0.00E+00		212316_at	NUP210	-0.57	3.00E-06
201885_s_at	CYB5R3	0.57	0.00E+00		231810_at	BRI3BP	-0.57	1.00E-06
225331_at	CCDC50	0.57	0.00E+00		211921_x_at	PTMA	-0.57	0.00E+00
225803_at	FBXO32	0.57	9.00E-06		241968_at	-	-0.56	0.00E+00
210757_x_at	DAB2	0.57	4.00E-06		200772_x_at	PTMA	-0.56	0.00E+00
201172_x_at	ATP6V0E1	0.57	2.00E-06		210053_at	TAF5	-0.56	1.00E-06
212119_at	RHOQ	0.57	0.00E+00		212479_s_at	RMND5A	-0.56	1.10E-05
200096_s_at	ATP6V0E1	0.57	0.00E+00		213947_s_at	NUP210	-0.55	2.00E-06
203827_at	WIPI1	0.56	3.00E-06		203209_at	RFC5	-0.55	6.00E-06
200885_at	RHOC	0.56	4.00E-06		224753_at	CDCA5	-0.55	1.30E-05
203167_at	TIMP2	0.56	1.00E-06		203856_at	VRK1	-0.55	9.00E-06
201279_s_at	DAB2	0.56	5.00E-06		202503_s_at	KIAA0101	-0.54	2.00E-06
200787_s_at	PEA15	0.56	0.00E+00		202387_at	BAG1	-0.54	6.00E-06
212120_at	RHOQ	0.56	0.00E+00		222264_at	HNRNPUL2	-0.54	2.00E-06
219637_at	ARMC9	0.56	1.00E-06		203302_at	DCK	-0.54	6.00E-06
212658_at	LHFPL2	0.55	9.00E-06		223154_at	MRPL1	-0.54	2.00E-06
202555_s_at	MYLK	0.55	0.00E+00		201969_at	NASP	-0.54	1.20E-05
212722_s_at	JMJD6	0.55	1.40E-05		202107_s_at	MCM2	-0.54	4.00E-06
227628_at	GPX8	0.55	0.00E+00		216384_x_at	LOC643287	-0.53	9.00E-06
200788_s_at	PEA15	0.55	0.00E+00		204026_s_at	ZWINT	-0.53	4.00E-06
231579_s_at	TIMP2	0.55	0.00E+00		228569_at	PAPOLA	-0.53	4.00E-06
228564_at	LOC375295	0.55	3.00E-06		223268_at	C11orf54	-0.53	0.00E+00
202134_s_at	WWTR1	0.55	1.00E-06		224760_at	SP1	-0.53	3.00E-06
200782_at	ANXA5	0.55	0.00E+00		222103_at	ATF1	-0.53	3.00E-06
224823_at	MYLK	0.54	9.00E-06		200047_s_at	YY1	-0.53	1.40E-05
211433_x_at	KIAA1539	0.54	1.80E-05		200091_s_at	RPS25	-0.53	3.00E-06
200663_at	CD63	0.54	5.00E-06		206687_s_at	PTPN6	-0.53	7.00E-06
218618_s_at	FNDC3B	0.54	6.00E-06		225716_at	-	-0.53	2.40E-05
231907_at	-	0.54	7.00E-06		215947_s_at	FAM136A	-0.53	2.00E-05
201278_at	DAB2	0.54	2.00E-05		215136_s_at	EXOSC8	-0.53	1.50E-05
209264_s_at	TSPAN4	0.53	3.00E-06		212482_at	RMND5A	-0.53	5.00E-06
208684_at	COPA	0.53	1.70E-05		218448_at	C20orf11	-0.53	1.00E-06
224560_at	TIMP2	0.53	0.00E+00		213054_at	-	-0.53	5.00E-06
203262_s_at	FAM50A	0.53	8.00E-06		235138_at	VPS35	-0.52	3.00E-06
200614_at	CLTC	0.53	8.00E-06		217956_s_at	ENOPH1	-0.52	4.50E-05
225032_at	FNDC3B	0.53	6.00E-06		219188_s_at	MACROD1	-0.52	4.10E-05
204140_at	TPST1	0.53	2.20E-05		227586_at	TMEM170A	-0.52	1.40E-05
201616_s_at	CALD1	0.53	1.10E-05		209421_at	MSH2	-0.52	1.10E-05
200700_s_at	KDELR2	0.53	1.00E-06		201930_at	MCM6	-0.52	3.40E-05
200770_s_at	LAMC1	0.53	1.00E-06		208808_s_at	HMGB2	-0.52	2.80E-05
219390_at	FKBP14	0.53	1.20E-05		202690_s_at	SNRPD1	-0.52	7.00E-06
226801_s_at	LOC646890	0.53	3.10E-05		201051_at	ANP32A	-0.52	1.10E-05
229017_s_at	DSTYK	0.53	3.00E-05		222214_at	-	-0.52	2.00E-06

214150_x_at	ATP6V0E1	0.52	2.20E-05		203633_at	CPT1A	-0.52	3.90E-05
212470_at	SPAG9	0.52	1.00E-06		204798_at	MYB	-0.51	0.00E+00
218717_s_at	LEPREL1	0.52	1.90E-05		209323_at	PRKRIR	-0.51	3.00E-05
202052_s_at	RAI14	0.52	7.00E-06		218458_at	GMCL1	-0.51	2.50E-05
200673_at	LAPTM4A	0.52	5.00E-06		202978_s_at	CREBZF	-0.51	2.90E-05
217785_s_at	YKT6	0.52	1.40E-05		208643_s_at	XRCC5	-0.51	2.80E-05
203891_s_at	DAPK3	0.52	2.30E-05		205393_s_at	CHEK1	-0.51	5.60E-05
223625_at	FAM126A	0.52	3.10E-05		200041_s_at	BAT1	-0.51	7.60E-05
201098_at	COPB2	0.52	2.60E-05		225887_at	C13orf23	-0.51	3.90E-05
212723_at	JMJD6	0.52	1.80E-05		211475_s_at	BAG1	-0.50	4.20E-05
210762_s_at	DLC1	0.52	1.40E-05		212138_at	PDS5A	-0.50	2.90E-05
204584_at	L1CAM	0.52	3.10E-05		201035_s_at	HADH	-0.50	7.00E-06
229465_s_at	PTPRS	0.52	3.10E-05		223711_s_at	THYN1	-0.50	4.50E-05
200643_at	HDLBP	0.52	1.40E-05		213762_x_at	RBMX	-0.50	5.00E-05
220990_s_at	MIR21	0.52	2.00E-06		208935_s_at	LGALS8	-0.50	1.30E-05
228776_at	GJC1	0.52	3.00E-06		200069_at	SART3	-0.50	3.20E-05
241763_s_at	FBXO32	0.51	4.40E-05		224610_at	SNHG1	-0.50	5.50E-05
225368_at	HIPK2	0.51	3.00E-06					
215489_x_at	HOMER3	0.51	0.00E+00					
224663_s_at	CFL2	0.51	0.00E+00					
212077_at	CALD1	0.51	8.00E-06					
204017_at	KDELR3	0.51	5.00E-06					
227239_at	FAM126A	0.51	2.20E-05					
200827_at	PLOD1	0.51	1.50E-05					
230146_s_at	FREQ	0.51	3.90E-05					
205717_x_at	PCDHGA12	0.51	2.60E-05					
209946_at	VEGFC	0.51	5.10E-05					
215706_x_at	ZYX	0.51	1.00E-06					
212667_at	SPARC	0.51	4.00E-05					
226893_at	-	0.51	3.10E-05					
200096_s_at	ATP6V0E1	0.51	3.30E-05					
202087_s_at	CTSL1	0.51	0.00E+00					
209263_x_at	TSPAN4	0.51	2.30E-05					
201536_at	DUSP3	0.51	8.00E-06					
226939_at	CPEB2	0.51	3.20E-05					
213112_s_at	SQSTM1	0.51	7.50E-05					
209079_x_at	PCDHGA12	0.51	2.50E-05					
211059_s_at	GOLGA2	0.51	3.10E-05					
208785_s_at	MAP1LC3B	0.50	2.20E-05					
200983_x_at	CD59	0.50	8.00E-06					
207765_s_at	KIAA1539	0.50	5.80E-05					
224791_at	ASAP1	0.50	3.30E-05					
212090_at	GRINA	0.50	1.10E-05					
200661_at	CTSA	0.50	1.00E-05					
225116_at	HIPK2	0.50	1.60E-05					
208782_at	FSTL1	0.50	4.90E-05					

Table S3: Correlation between the expression of proteins/phosphoproteins and the cell volume in the NCI60 cell lines.

Positively correlated				Negatively correlated		
Protein / Phosphoprotein	*PCC*	*p*		Protein / Phosphoprotein	*PCC*	*p*
Vimentin 57kD	0.36	0.0017		Phosphorylation of Cofilin at S3	-0.22	0.0589
AKT serine/threonine protein kinase	0.32	0.0055		E-cadherin protein	-0.20	0.0621
Phosphorylation of 4EBP1 (EIF4EBP1 Eukaryotic translation initiation factor 4E binding protein 1) protein at T37 and T46	0.29	0.0228		cleaved PARP (D214)	-0.20	0.0709
Phosphorylation of A-RAF at S299	0.28	0.0148		Phosphorylation of VEGFR2 at Y951	-0.19	0.0785
Phosphorylation of ATF2 at T69 and/or T71	0.26	0.0246		Phosphorylation of FRS2 alpha at Y436	-0.15	0.1554
Phosphorylation of ERK1/2 at T202 and/or Y204	0.21	0.0506		Phosphorylation of EGFR at Y1068	-0.14	0.1504
Bub3	0.20	0.0654		ErbB2 (receptor protein-tyrosine kinase)	-0.11	0.2049
Phosphorylation of FAK at Y397	0.20	0.0655		Phosphorylation of BCR at Y177	-0.11	0.2047
Phosphorylation of Aurora A at T288, Aurora B at T232 and/or Aurora C at T198	0.20	0.0514		Phosphorylation of ZAP70 at Y319 &/or SYK at Y352	-0.10	0.2212
Phosphorylation of CREB at S133	0.20	0.0624		Phosphorylation of EGFR at Y845	-0.10	0.2261
Phosphorylation of PKC alpha at S657	0.19	0.0683		Phosphorylation of Acetyl CoA carboxylase at S79	-0.10	0.2333
Phosphorylation of Arrestin 1 at S412	0.19	0.0826		Insulin Receptor beta	-0.09	0.2417
Cyclin D1 protein	0.17	0.0940		COX2 (PTGS2 Prostaglandin-endoperoxide synthase 2 (prostaglandin G/H synthase and cyclooxygenase)) protein	-0.07	0.2920
Phosphorylation of PKC alpha/betaII at T638 and/or T641	0.17	0.0917		Phosphorylation of PDGFR beta at Y751	-0.07	0.2821
Phosphorylation of Progesterone Receptor at S190	0.17	0.0990		Phosphorylation of Src protein at Y416	-0.07	0.2870
Phosphorylation of ASK1 at S83	0.17	0.1719		cleaved Caspase 3 (D175)	-0.06	0.3042
BMI1 (oncogene, DNA binding)	0.16	0.1173		Phosphorylation of LCK at Y505	-0.06	0.3335
ErbB3 (receptor protein-tyrosine kinase)	0.15	0.1091		Phosphorylation of TYK2 at Y1054 &/or Y1055	-0.06	0.3106
Phosphorylation of MARCKS at S152 and/or S156	0.15	0.1250		Phosphorylation of Src protein at Y527	-0.05	0.3227
4EBP-1 (EIF4EBP1 Eukaryotic translation initiation factor 4E binding protein 1) protein	0.14	0.1408		Phosphorylation of GAB1 at Y627	-0.05	0.3326
HIF-1alpha (Hypoxia inducible factor, basic helix-loop-helix transcription factor)	0.14	0.1461		Phosphorylation of CrkL at Y207	-0.05	0.3404
PDGFRb (pdgf-b receptor, tyrosine kinase)	0.14	0.1427		cleaved Caspase 6 (D162)	-0.05	0.3435
Phosphorylation of PKC pan/betaII at S660	0.14	0.1369		Phosphorylation of SHIP1 at Y1020	-0.05	0.3223
Phosphorylation of AKT on Ser473	0.14	0.1496		Phosphorylation of SHC at Y317	-0.05	0.3372
Phosphorylation of Bcl-2 at T56	0.14	0.1522		Phosphorylation of STAT3 protein at T727	-0.04	0.3469
Phosphorylation of TSC2 (Tuberous sclerosis 2) protein at T1462	0.13	0.1494		cleaved Caspase 7 (D198)	-0.04	0.3238
Phosphorylation of elF4G at S1108	0.13	0.1627		Phosphorylation of p38 (MAPK14 Mitogen-activated protein kinase 14) protein at T180/Y182	-0.04	0.3140
Phosphorylation of NFkB p65 at S536	0.13	0.1718		Phosphorylation of STAT6 (Signal transducer and activator of transcription 6) protein at Y641	-0.04	0.3404
Epidermal growth factor receptor	0.12	0.1893		PLK1, polo-like kinase 1	-0.04	0.3355
Phosphorylation of Histone H3 at S28	0.11	0.1996		Phosphorylation of VEGFR2 at Y996	-0.04	0.3375
Phosphorylation of Ask1 at Y284	0.11	0.2043		Phosphorylation of PLC gamma1 at Y783	-0.03	0.3640
SMAD 1/5/8 (S/S)	0.11	0.2098		Phosphorylation of SMAD2 at S465 &/or 467	-0.03	0.3503
Phosphorylation of ATP Cytrate Lyase at S454	0.11	0.2135		Phosphorylation of ErbB2 at Y1248	-0.03	0.3631
Bcl-2 (Apoptosis regulator)	0.10	0.2309		cleaved Lamin A (D230)	-0.03	0.3845
Phosphorylation of FKHR at T24 and/or FKHRL1 at T32	0.10	0.2320		Phosphorylation of EGFR at Y1173	-0.03	0.3480
Phosphorylation of Adducin at S662	0.09	0.2550		Phosphorylation of c-Abl of Y245	-0.02	0.3742
Phosphorylation of RPS6 (Ribosomal protein S6) protein at S235 and S236	0.09	0.2543		Phosphorylation of MAPK8 (JNK, mitogen-activated protein kinase 8) protein at T183/Y185	-0.02	0.3715
Phosphorylation of 4EBP1 (EIF4EBP1 Eukaryotic translation initiation factor 4E binding protein 1) protein at T70	0.09	0.2655		Phosphorylation of Histone H3 at S10, mitosis maker	-0.02	0.3729
Phosphorylation of EGFR at Y1045	0.09	0.2657		Phosphorylation of PDGFR beta at Y716	-0.02	0.3744
PTEN protein-tyrosine phosphatase	0.09	0.2684		Phosphorylation of RET at Y905	-0.02	0.4118
Phosphorylation of EGFR at S1046 and/or S1047	0.09	0.2663		Phosphorylation of BAD (BCL2-associated agonist of cell death) protein at S112	-0.02	0.4311
Phosphorylation of eNOS-NOSIII at S116	0.08	0.2882		Phosphorylation of FAK at Y576 and/or 577	-0.02	0.3685
Phosphorylation of RPS6 (Ribosomal protein S6) protein at S240 and S244	0.08	0.2754		Phosphorylation of Raf at S259	-0.02	0.3866

Phosphorylation of EGFR at Y992	0.08	0.2875		Phosphorylation of SMAD2 at S245 &/or S250 &/or S255	-0.02	0.3825
signal transducer and activator of transcription 3 (acute-phase response factor)	0.08	0.2875		Smac/Diablo	-0.02	0.3914
Phosphorylation of CHK1 at S345	0.08	0.2773		CASP9, caspase 9, apoptosis-related cysteine protease	-0.01	0.3962
Phosphorylation of AMPK alpha1 at S485	0.08	0.2985		p38 (MAPK14 Mitogen-activated protein kinase 14) protein	-0.01	0.3962
Phosphorylation of c-Kit at Y703	0.08	0.2894		p70S6K (RPS6KB1 Ribosomal protein S6 kinase, 70kDa, polypeptide 1) protein	-0.01	0.4111
Phosphorylation of EGFR at Y1148	0.08	0.3155		Phosphorylation of PKC zeta-lambda at T410 and/or T403	-0.01	0.3913
Phosphorylation of p53 at S15	0.07	0.3052		Phosphorylation of MET at Y1234 and/or Y1235	-0.01	0.3890
Phosphorylation of AMPK beta1 at S108	0.07	0.3245		cyclin B1	-0.01	0.4391
Phosphorylation of PRAS40 at T246	0.07	0.3321		Phosphorylation of PAK1 at S199 and/or S204 and/or PAK2 at S192 and/or S197	-0.01	0.4037
Phosphorylation of 4EBP1 (EIF4EBP1 Eukaryotic translation initiation factor 4E binding protein 1) protein at S65	0.07	0.3290		Phosphorylation of PYK2 at Y402	-0.01	0.4515
Phosphorylation of FKHR at S256	0.07	0.3330		Phosphorylation of ALK at Y1586	-0.01	0.4550
Phosphorylation of T308 on AKT	0.07	0.3088		Phosphorylation of PKC theta at T538	-0.01	0.4044
ER (estrogen receptor) alpha protein	0.07	0.3014		cleaved Caspase 9 (D315)	-0.01	0.4031
Phosphorylation of mTOR (Mechanistic target of rapamycin (serine/threonine kinase)) protein at S2448	0.07	0.3453				
Phosphorylation of IkB alpha at S32 and/or S36	0.06	0.3633				
Phosphorylation of c-Kit at Y719	0.06	0.3537				
Cyclin A (Cell cycle regulation)	0.06	0.3267				
Phosphorylation of CrkII at Y221	0.05	0.3595				
Phosphorylation of FADD at S194	0.05	0.3920				
Phosphorylation of ATF2 at T71	0.05	0.3677				
Phosphorylation of Estrogen Receptor alpha at S118	0.05	0.4112				
Phosphorylation of PDK1 (PDPK1 3-phosphoinositide dependent protein kinase-1) protein at S241	0.05	0.4023				
Beclin1	0.05	0.3848				
Phosphorylation of p27 of T187	0.04	0.4362				
Phosphorylation of IRS1 at S612	0.04	0.4369				
Phosphorylation of SGK1 (Serum/glucocorticoid regulated kinase 1) protein at S78	0.04	0.4444				
Phosphorylation of B-Raf at S445	0.04	0.4044				
MEK 1/2 protein	0.04	0.4386				
Survivin	0.04	0.4370				
Phosphorylation of MEK1 and MEK2 proteins at S217/S221	0.04	0.4564				
Phosphorylation of PTEN at S380	0.04	0.4558				
Phosphorylation of Syk at Y525 &/or Y526	0.03	0.4711				
p53	0.03	0.4024				
Phosphorylation of RSK3 at T356 and/or S360	0.03	0.4580				
IRS1 (insulin receptor substrate 1) protein	0.03	0.4722				
Phosphorylation of C-RAF at S338	0.03	0.4839				
mTOR (Mechanistic target of rapamycin (serine/threonine kinase)) protein	0.03	0.4749				
Phosphorylation of eNOS at S113	0.03	0.4574				
Phosphorylation of BAD at S155	0.03	0.4776				
Phosphorylation of IGF-1 Receptor at Y1135/1136/Insulin Receptor (Y1150/1151)	0.03	0.4101				
Phosphorylation of PRK1 at T774 and/or PRK2 at T816	0.03	0.4680				
Phosphorylation of Chk2 at S33 and/or S35	0.03	0.4109				
Phosphorylation of BAD at S136	0.03	0.4715				
Phosphorylation of p70 S6 Kinase at S371	0.03	0.5001				
Phosphorylation of ErbB3 at Y1197	0.03	0.5001				
Phosphorylation of SHP2 at Y580	0.03	0.5081				
Phosphorylation of Bcl-2 at S70	0.03	0.5007				
CREB protein	0.02	0.5019				
Phosphorylation of RAS-GFR1 at S916	0.02	0.5036				
Bad (BCL2-antagonist of cell death)	0.02	0.4822				
Phosphorylation of SEK1-MKK4 at S80	0.02	0.5118				
Phosphorylation of PKC delta at T505	0.02	0.5229				
Phosphorylation of p90RSK at S380	0.02	0.4965				
Bax (BCL2-associated X protein)	0.02	0.5022				

Phosphorylation of eNOS at S1177	0.02	0.4352				
X-IAP (x-linked inhibitor of apoptosis)	0.02	0.5139				
Phosphorylation of IGF1 Receptor at Y1131 and/or Insulin Receptor at Y1146	0.02	0.5183				
Phosphorylation of LKB1 at S334	0.02	0.5210				
Phosphorylation of STAT3 protein at Y705	0.02	0.4512				
Phosphorylation of p40 phox at T154	0.02	0.4772				
Phosphorylation of c-Abl of T735	0.02	0.5289				
Phosphorylation of mTOR at S2481	0.02	0.5280				
Phosphorylation of VEGFR2 at Y1175	0.02	0.5296				
Phosphorylation of PAK1 at T423 and/or PAK2 at T402	0.02	0.5498				
Phosphorylation of Mst1 at T183 and/or Mst 2 at T180	0.02	0.5401				
Phosphorylation of MSK1 at S360	0.01	0.5391				
Phosphorylation of ErbB3 at Y1289	0.01	0.5420				
Bak (BCL2-antagonist/killer 1)	0.01	0.5019				
Phosphorylation of PLK1 at T210	0.01	0.5580				
Phosphorylation of Jak2 at Y1007 and/or Y1008	0.01	0.5496				
Phosphorylation of ACK1 at Y857 and/or Y858	0.01	0.5495				
Phosphorylation of TUBERIN TSC2 at Y1571	0.01	0.5516				
ErbB4 (receptor protein-tyrosine kinase)	0.01	0.5462				
Phosphorylation of FKHRL1 at S253	0.01	0.5613				
Phosphorylation of beta Catenin at T41 and/or S45	0.01	0.5414				
Phosphorylation of p70 S6 Kinase at T412	0.01	0.5469				
Phosphorylation of cPLA2 at S505	0.01	0.5530				
Phosphorylation of PKA C at T197	0.01	0.5570				
Phosphorylation of LKB1 (STK11 serine/threonine kinase 11) protein at S428	0.01	0.5597				
Phosphorylation of Ezrin at T156, Radixin at T564, and/or Moesin at T558	0.01	0.5664				
Phosphorylation of STAT2 at Y690	0.01	0.5411				
Phosphorylation of p70S6K (RPS6KB1 Ribosomal protein S6 kinase, 70kDa, polypeptide 1) protein at T389	0.01	0.5607				
Phosphorylation of Paxillin at Y118	0.00	0.5514				
Phosphorylation of Elk1 at S383	0.00	0.5751				
cleaved Caspase 9 (D330)	0.00	0.5631				
Phosphorylation of GSK3 alpha at Y279 and/or GSK3 beta at Y216	0.00	0.5722				
Phosphorylation of eIF4E at S209	0.00	0.4562				
Phosphorylation of JAK1 at Y1022 and/or Y1023	0.00	0.4194				
KIT (receptor for stem cell factor, tyrosine kinase)	0.00	0.5002				
Phosphorylation of GSK3 alpha/beta at S21and or S9	0.00	0.4331				
ERK 1/2 protein	0.00	0.4224				
Phosphorylation of STAT5 protein at Y694	0.00	0.4802				
Phosphorylation of STAT1 at Y701	0.00	0.4367				
Bcl-xl (BCL2-like 1)	0.00	0.4249				
Phosphorylation of VAV3 at Y173	0.00	0.4066				

Table S4: FDA approved drugs included in the *in vitro* growth inhibition analysis. NSC stands for National Service Center and it is the identifier utilized by the Department of Therapeutics Program at the National Cancer Institute [22].

NSC	Mechanism of Action	Name
177023	Alkaline Phosphatase Inhivitor	Levamisole
13875	Alkylating Agents	Altretamine
409962	Alkylating Agents	BCNU
125066	Alkylating Agents	Bleomycin
750	Alkylating Agents	Busulfan
241240	Alkylating Agents	Carboplatin
3088	Alkylating Agents	Chlorambucil
119875	Alkylating Agents	Cisplatin
26271	Alkylating Agents	Cyclophosphamide
45388	Alkylating Agents	Dacarbazine
109724	Alkylating Agents	Ifosfamide
79037	Alkylating Agents	Lomustine
8806	Alkylating Agents	Melphalan
26980	Alkylating Agents	Mitomycin C
762	Alkylating Agents	Nitrogen mustard
266046	Alkylating Agents	Oxaliplatin
25154	Alkylating Agents	Pipobroman
6396	Alkylating Agents	Thiotepa
9706	Alkylating Agents	Tretamin
34462	Alkylating Agents	Uracil mustard
628503	Antimitotic Agents	Docetaxel
608210	Antimitotic Agents	Navelbine
125973	Antimitotic Agents	Taxol
49842	Antimitotic Agents	Vinblastine
67574	Antimitotic Agents	Vincristine
719344	Aromatase Inhibitor	Anastrozole
713563	Aromatase Inhibitor	Exemestane
719345	Aromatase Inhibitor	Letrozole
23759	Aromatase Inhibitor	Testolactone
719627	COX-2 Inhibitor	Celebrex
27640	DNA Antimetabolites	5-Fluorouracil deoxyriboside
63878	DNA Antimetabolites	Ara-C
287459	DNA Antimetabolites	Cytarabine
127716	DNA Antimetabolites	Decitabine
312887	DNA Antimetabolites	Fludarabine phosphate
613327	DNA Antimetabolites	Gemcitabine
32065	DNA Antimetabolites	Hydroxyurea
218321	DNA Antimetabolites	Pentostatin
752	DNA Antimetabolites	Thioguanine
755	DNA Antimetabolites	Thiopurine
71423	Estrogen analog	Ethinyl Estradiol
719276	Estrogen Receptor Antagonist	Fulvestrant
180973	Estrogen Receptor Antagonist	Tamoxifen
226080	mTOR inhibitor	Rapamycin
683864	mTOR inhibitors	Temsirolimus
733504	mTOR inhibitors	Everolimus
105014	DNA Antimetabolites	Cladribine
686673	NA	2-Amino-6-methoxypurine arabinoside
713563	NA	6-Methylenandrosta-1,4-diene-3,17-dione
14229	NA	Acrichine
369100	NA	Aldara
246131	NA	Antibiotic AD 32
92859	NA	Arsenic Oxide, As2O3
706363	NA	Arsenic Trioxide

1390	NA	Bleminol
3590	NA	Calcium folinate
88536	NA	Calusterone
38721	NA	Chloditan
702294	NA	Estramustine sodium phosphate
296961	NA	Ethiofos
12198	NA	Masterone/Econazole
45923	NA	Meladinine
279836	Topoisomerase II Inhibitors	Mitoxantrone
23162	NA	Nandrolin
77213	NA	Natunalar
18509	NA	Pentanoic acid, 5-amino-4-oxo-, hydrochloride
256942	NA	Pidorubicin hydrochloride
122758	NA	Retinoic acid
701852	NA	SAHA
362856	NA	Temozolomide
113891	NA	Uromitexan
66847	NA	Yodomin
85998	O-GlcNAc Inhibitor	Streptozocin
681239	Proteasome Inhibitor	Bortezomib
698037	RNA/DNA Antimetabolites	Pemetrexed
102816	RNA/DNA Antimetabolites	Azacytidine
712807	RNA/DNA Antimetabolites	Capecitabine
606869	RNA/DNA Antimetabolites	Clofarabine
19893	RNA/DNA Antimetabolites	Fluorouracil
740	RNA/DNA Antimetabolites	Methotrexate
82151	Topoisomerase II Inhibitors	Daunorubicin
123127	Topoisomerase II Inhibitors	Doxorubicin
141540	Topoisomerase II Inhibitors	Etoposide
169780	Topoisomerase II Inhibitors	ICRF-187
256439	Topoisomerase II Inhibitors	Idarubicin
122819	Topoisomerase II Inhibitors	Teniposide
616348	Topoisomerase I Inhibitors	Irinotecan
609699	Topoisomerase I Inhibitors	Topotecan
3053	Transcription/DNA synthesis Inhibitor	Actinomycin D
24559	Transcription/DNA synthesis Inhibitor	Mithramycin
697979	Translation Inhibitor	Ontak
732517	Tyrosine Kinase Inhibitor	Dasatinib
718781	Tyrosine Kinase Inhibitor	Erlotinib
715055	Tyrosine Kinase Inhibitor	Gefitinib
688097	Tyrosine Kinase Inhibitor	Herceptin
743414	Tyrosine Kinase Inhibitor	Imatinib
632307	Tyrosine Kinase Inhibitor	Lapatinib
747599	Tyrosine Kinase Inhibitor	Nilotinib
747971	Tyrosine Kinase Inhibitor	Sorafenib
750690	Tyrosine Kinase Inhibitor	Sunitinib
633782	Sterol Synthesis Inhibitor	Simvastatin
633781	Sterol Synthesis Inhibitor	Lovastatin
281245	Sterol Synthesis Inhibitor	Mevastatin

Cell line	Protein synthesis rate (fmol/hour/cell)	Protein content (ng/cell)
BR:BT-549	89	0.262
BR:HS578T	128	0.295
BR:MCF7	70	0.185
BR:MDA-MB-231	47	0.241
BR:T-47D	52	0.253
CNS:SF-268	55	0.308
CNS:SF-295	285	0.411
CNS:SF-539	90	0.394
CNS:SNB-19	111	0.326
CNS:SNB-75	77	0.786
CNS:U251	78	0.361
CO:COLO205	64	0.257
CO:HCC-2998	125	0.279
CO:HCT-116	114	0.202
CO:HCT-15	69	0.179
CO:HT29	99	0.250
CO:KM12	35	0.198
CO:SW-620	61	0.132
LC:A549/ATCC	74	0.244
LC:EKVX	55	0.413
LC:HOP-62	73	0.389
LC:HOP-92	128	0.560
LC:NCI-H226	65	0.550
LC:NCI-H23	50	0.378
LC:NCI-H322M	73	0.221
LC:NCI-H460	57	0.322
LC:NCI-H522	67	0.465
LE:CCRF-CEM	43	0.076
LE:HL-60	62	0.162
LE:K-562	133	0.231
LE:MOLT-4	33	0.101
LE:RPMI-8226	139	0.167
LE:SR	45	0.021
ME:LOXIMVI	124	0.283
ME:M14	82	0.259
ME:MALME-3M	61	0.319
ME:MDA-MB-435	90	0.211
ME:MDA-N	NA	NA
ME:SK-MEL-2	143	0.301
ME:SK-MEL-28	181	0.345
ME:SK-MEL-5	191	0.294
ME:UACC-257	49	0.254
ME:UACC-62	107	0.664
OV:IGROV1	124	0.308
OV:OVCAR-3	60	0.257
OV:OVCAR-4	65	0.441
OV:OVCAR-5	35	0.230
OV:OVCAR-8	34	0.234
OV:OVCAR-8/ADR	51	0.285
OV:SK-OV-3	56	0.257
PR:DU-145	58	0.390
PR:PC-3	154	0.606
RE:786-0	79	0.372
RE:A498	97	0.426
RE:ACHN	155	0.279
RE:CAKI-1	101	0.555
RE:RXF-393	117	0.581
RE:SN12C	62	0.214
RE:TK-10	46	0.373
RE:UO-31	46	0.215

Table S5: MLE protein synthesis rates and measured protein content for each NCI60 cell line.

Amino acid	σ_a (fmol/cell/hour)
isoleucine	2.0
leucine	2.1
lysine	2.5
methionine	2.6
phenylalanine	2.6
threonine	2.8
tryptophan	2.9
valine	2.9

Table S6: Maximum likelihood estimate of the variances (σ_a). The value for histidine is not shown because its exchange fluxes were not reported.
